# Proteomic Analysis of the Secretome and Exosomes of Feline Adipose-Derived Mesenchymal Stem Cells

**DOI:** 10.3390/ani11020295

**Published:** 2021-01-24

**Authors:** Antonio J. Villatoro, María del Carmen Martín-Astorga, Cristina Alcoholado, María del Mar Sánchez-Martín, José Becerra

**Affiliations:** 1 Laboratory of Bioengineering and Tissue Regeneration, Department of Cell Biology, Genetics and Physiology, University of Málaga, IBIMA, 29071 Málaga, Spain; antoniojvillatoro@gmail.com (A.J.V.); mcmartinastorga@gmail.com (M.d.C.M.-A.); alcoholado.c@gmail.com (C.A.); mdm.sanchez@outlook.com (M.d.M.S.-M.); 2 Instituto de Immunología Clínica y Terapia Celular (IMMUNESTEM), 29071 Málaga, Spain; 3 Biomedical Research Networking Center in Bioengineering, Biomaterials, and Nanomedicine (CIBER-BBN), 28029 Madrid, Spain; 4 Andalusian Centre for Nanomedicine and Biotechnology, University of Málaga (BIONAND), 29590 Málaga, Spain

**Keywords:** mesenchymal stem cells, feline, exosomes, secretome, ultrahigh-performance liquid chromatography-high-resolution mass spectrometry (UHPLC–HRMS)

## Abstract

**Simple Summary:**

The enormous advances in stem cell research have generated high expectations in the development of new therapies to repair or regenerate damaged tissues. For this reason, laboratory studies of stem cells enable scientists to learn about cells’ essential properties. Specifically, in recent years, therapies based on mesenchymal stem cells have become an interesting alternative for the treatment of different complex pathologies in veterinary medicine. Mesenchymal stem cells secrete a wide variety of therapeutic elements such as bioactive molecules and extracellular vesicles (e.g., exosomes). Thus, it is essential to characterize them before future use as biotechnological products. Therefore, the objective of this study was to determine and compare their protein profile to understand better the mechanisms of action of these components and facilitate their possible use in future therapies. The data demonstrate the existence of different proteins responsible for the biological effects of cells. In addition, these approaches and techniques can contribute to the better prediction of clinical outcomes of mesenchymal stem cell treatment.

**Abstract:**

Mesenchymal stem cells (MSCs) have been shown to have therapeutic efficacy in different complex pathologies in feline species. This effect is attributed to the secretion of a wide variety of bioactive molecules and extracellular vesicles, such as exosomes, with significant paracrine activity, encompassed under the concept of the secretome. However, at present, the exosomes from feline MSCs have not yet been studied in detail. The objective of this study is to analyze and compare the protein profiles of the secretome as a whole and its exosomal fraction from feline adipose-derived MSCs (fAd-MSCs). For this, Gene Ontology (GO), Kyoto Encyclopedia of Genes and Genomes (KEGG) and Protein–Protein Interaction Networks Functional Enrichment Analysis (STRING) were utilized. A total of 239 proteins were identified in the secretome, and 228 proteins specific to exosomes were identified, with a total of 133 common proteins. The proteins identified in the secretome were located in the extracellular regions and in the cytoplasm, while the exosomal proteins were located mainly in the membrane, cytoplasm and cytosol. Regarding function, in the secretome, proteins involved in different metabolic pathways, in pathways related to the immune system and the endocrine system and in the processing of proteins in the endoplasmic reticulum predominated. In contrast, proteins specific to exosomes were predominantly associated with endocytosis, cell junctions, platelet activation and other cell signaling pathways. The possible future use of the secretome, or some of its components, such as exosomes, would provide a non-cell-based therapeutic strategy for the treatment of different diseases that would avoid the drawbacks of cell therapy.

## 1. Introduction

In recent years, mesenchymal stem cells (MSCs) therapy has become an interesting alternative in different complex pathologies in feline medicine [1,2,3]. Feline MSCs were first isolated from different sources such as bone marrow [4], fat [5], peripheral blood [6], and fetal fluids and amniotic membranes [7]. Currently, adipose-derived MSCs are the most used in clinical applications in feline species due to their ease of obtaining, affordability, and abundance [3,5,6,7,8,9,10,11,12,13].

The therapeutic effect of MSCs is attributed to the secretion of a wide variety of bioactive molecules and extracellular vesicles with important paracrine activity, encompassed under the concept of the secretome [14,15,16]. Among extracellular vesicles, exosomes are of great interest as therapeutic elements. Exosomes are nanovesicles of endocytic origin with a bilipid membrane that transport different types of proteins, lipids, and microRNAs, among other molecules. Their role is influenced by the cellular origin and different physiological and pathological conditions, and they play a fundamental role in intercellular communication [17,18,19].

Future use of the secretome or some of its components, such as exosomes, as biotechnological products in regenerative medicine, provides the basis for a cell-free therapeutic strategy that avoids some drawbacks of cell therapy [20,21,22]. They have shown their efficacy in a wide variety of pathologies, mainly in the human species [23,24,25,26] but studies in veterinary medicine are still scarce [22,27,28,29]. Therefore, it is essential to characterize the secretome and its components before their possible clinical use.

Although exosomes have been characterized in different types of MSCs, both in human species and in some domestic animals [17,30,31,32], to date, exosomes in MSCs of feline species have not been described in detail.

Considering that proteins mediate most intracellular and intercellular activities, approaches in proteomics are of great value to elucidate different cellular functions and possible patterns of cellular communication [33,34,35]. Therefore, the characterization of the proteomic profile of the MSCs secretome under standard culture conditions, as well as that of MSCs exosomes, will allow a better understanding of the mechanisms of action of these components and facilitate their possible use in future therapies.

Ongoing research on feline MSCs confirms that they also have an immunomodulatory potency similar to that most recently defined for human MSCs [2,16]. Since the cat is an interesting preclinical model that naturally suffers from certain human-like pathologies, the translation of its results with these new therapies should be very useful in human medicine [36].

Therefore, the objective of this study was to determine and compare the protein profile of the secretome with that of exosomes contained therein that were previously isolated, quantified and characterized from feline adipose-derived MSCs (fAd-MSCs) under standard culture conditions.

## 2. Materials and Methods

All protocols were approved by the Institutional Committee for the Care and Use of Animals of BIONAND (Centro Andaluz de Nanomedicina y Biotecnología), Malaga, Spain with the project identification code 07/2017. The procedures with animals were performed by veterinarians after obtaining consent from the owners for the transfer of samples.

### 2.1. Isolation, Expansion and Characterization of fAd-MSCs

fAd-MSCs were isolated from subcutaneous fat of the inguinal region of three healthy, European breed female cats, aged between 3 and 6 months; the fat samples were obtained at the time of ovariectomy. The adipose tissue was digested with collagenase type I (Merck) to separate the adipose fraction from the vascular stroma. The isolated fraction was seeded in Dulbecco’s modified Eagle’s medium (DMEM) supplemented with 10% fetal bovine serum (FBS), 2.5 mM L-glutamine, 100 U/mL penicillin, 100 µg/mL streptomycin, and 1.25 µg/mL fungizone (Merck) in T-175 flasks. The fAd-MSCs were maintained under standard culture conditions until reaching a confluence of 80%, at which time they were subcultured. All experiments were performed in cells of culture passage 2.

fAd-MSCs were characterized by flow cytometry and the multipotential differentiation capacity into adipogenic, osteogenic and chondrogenic lineages, according to the criteria of the International Society of Cellular Therapy (ISCT) [37,38]. The detailed procedure was published previously by our group [3].

### 2.2. Production of the fAd-MSC Secretome

Each donor was processed individually. fAd-MSCs (5 × 10^5^) were seeded in DMEM supplemented with 10% FBS, 2.5 mM L-glutamine, 100 U/mL penicillin, and 100 µg/mL streptomycin in n T-75 flask. The culture medium was changed twice a week until reaching 80% confluence. At this time, two washes with PBS were performed, and 15 mL of DMEM without supplements (D1145, Sigma) was added to each flask. The flasks were placed in an incubator for 24 h, and then, the medium was collected and filtered using a 0.22-µm filter to isolate the secretome; the secretome was stored at −80 °C until use. The cells present at the time the medium was removed were counted using a Neubauer chamber, and their viability was evaluated with trypan blue. Subsequently, an equitable secretome mixture from each donor was lyophilized (LyoQuest, TELSTAR) and reconstituted in 500 μL of sterile bidistilled water to be analyzed in a mass spectrometer.

### 2.3. Isolation of Exosomes from fAd-MSCs

The isolation of exosomes from fAd-MSCs was performed by ultracentrifugation, following the protocol previously used by our team [17]. In summary, 3 T-175 flasks for each donor were seeded with 1 × 10^6^ fAd-MSCs in DMEM supplemented with 10% FBS, 2.5 mM L-glutamine, 100 U/mL penicillin, and 100 µg/mL streptomycin. Upon reaching 80% confluence, the cells were washed with PBS, and DMEM supplemented with 10% exosome-free FBS was added. Exosome-free FBS was previously obtained by ultracentrifugation (100,000× *g* for 60 min at 4 °C). The culture medium was removed after 72 h of incubation under standard conditions. The cells were counted in a Neubauer chamber, and their viability was evaluated with trypan blue.

Subsequently, the culture medium was centrifuged at 13,000× *g* for 30 min at 4 °C to remove cell debris and microvesicles. Then, it was ultracentrifuged using a 70 Ti fixed-angle rotor in an Optima LE-80K ultracentrifuge (Beckman Coulter) at 100,000× *g* for 60 min at 4 °C to sediment the exosomes. The exosomes were resuspended in PBS and recentrifuged under the same conditions as above. Finally, the exosomes were resuspended in 100 µL of PBS, and the protein concentrations were quantified using a BCA kit (Thermo Scientific); the exosomes were stored at −80 °C until use. Each sample was processed individually, and for characterization, an equitable mixture for each donor was used.

### 2.4. Transmission Electron Microscopy

A 20 μL sample of exosomes, previously diluted with distilled water, was placed on a carbon-coated nickel grid (Aname) and allowed to dry overnight at room temperature. The samples were analyzed at different magnifications using a transmission electron microscope (Morgagni 268D, Philips) according to instructions provided by the Transmission Electron Microscopy Unit of the Central Research Support Service (Servicio Central de Apoyo a la Investigación), SCAI, UMA [39].

### 2.5. Size Distribution and Electronegativity of Exosomes

The size, distribution and zeta potential of the exosomes were analyzed using a Malvern Zetasizer 2000 (Malvern Instruments) after diluting the samples in 1 mL of distilled water. The Zetasizer was used to determine the size distribution of the exosome population in nanometers. In addition, the homogeneity (or polydispersity) of the sample was determined. Each sample was measured in triplicate, and the results are expressed as the mean value ± standard deviation [39].

### 2.6. Expression of Exosomal Markers

Exosome-specific markers were detected by western blot analysis (WB) [19,40]. Thirty micrograms of exosomes were used to perform SDS-PAGE, followed by semidry transfer. The following primary antibodies were used (overnight incubation): rabbit anti-ALIX (Abcam), rabbit anti-Tsg101 (Abcam), rabbit anti-calnexin (Cell Signaling Technology) and mouse anti-Hsp70 (Santa Cruz Biotechnology). The following peroxidase-conjugated antibodies were used: anti-rabbit (Abcam) and anti-mouse (Merck). The membranes were incubated in ECL (Cell Signaling Technology). Proteins were visualized using a ChemiDocTM XRS + system (BioRad). Protein lysate of human adipose MSCs were used as a positive control.

### 2.7. Proteomic Analysis by UHPLC-HRMS

The secretome and exosome protein profiles were analyzed by the Proteomics Service of the SCAI of the Universidad de Málaga (University of Malaga) [17]. The proteins in the samples were purified by a precipitation procedure with modified trichloroacetic acid (Clean-Up Kit; GE Healthcare, München, Germany). Next, gel-assisted proteolysis was carried out by trapping the protein solution in a polyacrylamide gel matrix. The proteins were reduced, and their cysteine residues were carbamidomethylated and digested with trypsin (Promega, Madison, WI, USA). Peptides were extracted from the gel, purified and concentrated using a C18 ZipTip (Merck Millipore) according to the manufacturer’s instructions.

The samples were injected into an Easy nLC 1200 UHPLC system coupled to a quadrupole-Orbitrap hybrid mass spectrometer (Q Exactive ^TM^ HF-X, Thermo Fisher Scientific). Data were acquired using Tune 2.9 and Xcalibur 4.1 software (Thermo Fisher Scientific). The peptides were automatically loaded on a preanalytical column (Acclaim PepMap 100, 75 µm × 2 cm, C18, 3 µm, 100 A, Thermo Fisher Scientific) and eluted on a 50-cm analytical column (PepMap RSLC C18, 2 µm, 100 A, 75 µm × 50 cm, Thermo Fisher Scientific). The binary gradient mobile phase consisted of 0.1% formic acid in water (solvent A) and 0.1% formic acid in 80% acetonitrile (solvent B). The peptides were eluted from the analytical column with a 120 min gradient ranging from 2% to 20% solvent B, followed by a 30 min gradient ranging from 20% to 35% solvent B and finally 15 min of 95% solvent B at a constant flow rate of 300 nL/min.

Data acquisition was performed in the positive ionization mode. The MS1 spectra were acquired in a range of 300–1750 *m/z* at a resolution of 120,000. The precursor ions were isolated within a 1.2 *m/z* window using a data-dependent acquisition method and fragmented to obtain the MS/MS spectra corresponding to a resolution of 30,000.

Raw data were analyzed using Proteome DiscovererTM 2.2 (Thermo Scientific). For the identification of the MS/MS spectra, the Sequest HT search engine and the NCBI Felis catus protein database version 2017.10.30 (43,896 sequences) were used as search engines. Protein assignments were validated using the Percolator^®^ algorithm [41] imposing a strict 1% false-positive rate (FDR) limit. The results were filtered to accept only those proteins with at least two identified peptide sequences.

### 2.8. Bioinformatic Analysis

The secretome and exosome proteins from the standard culture of fAd-MSCs were subjected to bioinformatic analysis to classify them according to genetic ontology, to identify the metabolic pathways involved and to highlight the protein–protein interaction networks. For this, the identified sequences were mapped using Proteome Discoverer 2.2 (Thermo Fisher Scientific, Waltham, MA, USA) according to the biological processes, molecular functions and cellular components provided by the Gene Ontology (GO) database [42,43]. In addition, STRING (v.11) [44] was used to determine possible interactions between differentially expressed proteins. STRING tracks both direct (physical) and indirect (functional) protein–protein interactions, both theoretical and experimental, collected from sources such as the genomic context, experimental and coexpression data and previous information. The Felis catus database was used for all STRING analyses. Each node represented a protein, and each link represented an interaction. The interactions included physical and functional associations. STRING and Cytoscape (v. 3.8.0) were used with the MCODE plugin (v. 1.6.1) [45] to visualize the clusters or highly interconnected regions of the network. Functional enrichment was determined using the Kyoto Encyclopedia of Genes and Genomes (KEGG) database [46]. Specifically, KEGG routes with average specificity and a kappa score of 0.4 were explored. An enrichment/depletion method was applied with a two-sided hypergeometric test, corrected with Bonferroni reduction for each p-value calculation. Enriched pathways with a value of *p* < 0.05 were considered significant.

## 3. Results

### 3.1. Characterization of fAd-MSCs

The isolated fAd-MSCs were a homogeneous cell population positive for the mesenchymal markers CD29, CD44, CD73, CD90, and MHC-I and negative for the hematopoietic markers CD34, CD45 and MHC-II. The fAd-MSCs differentiated in the three mesodermal lineages after appropriate induction; adipogenic, confirmed by the existence of Oil-red-O-positive fat drops, osteogenic, confirmed by alizarin red-positive calcium deposits, and chondrogenic differentiation, confirmed by micromasses that exhibited metachromasia when stained with toluidine blue.

### 3.2. Characterization of the Exosomes of fAd-MSCs

**Figure 1:**
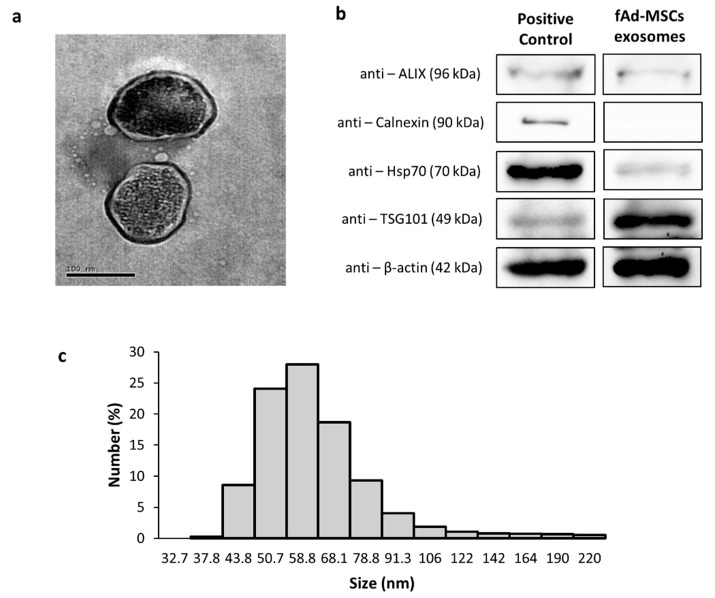
Characterization of the exosomes of feline adipose-derived mesenchymal stem cells (fAd-MSCs). (**a**) Image of exosomes examined by transmission electron microscopy. Bars: 100 nm; (**b**) Western blot analysis shows a positive expression of specific exosomal markers; (**c**) Exosome size distribution.

The exosomes of fAd-MSCs were examined by TEM and dynamic light scattering (DLS). The DLS results indicated that the exosomes had diameters ranging from 30 to 200 nm and a Z potential of −17.37 ± 1.0 mV. The polydispersity index (0.385) confirmed homogeneity in the samples (Figure 1A,C). The fAd-MSCs exosomes expressed exosomal-specific markers, such as ALIX, heat shock protein 70 (Hsp70) and tumor susceptibility gene 101 (TSG101), which are involved in exosomal biogenesis and vesicular trafficking (Figure 1B), but did not express calnexin (endoplasmic reticulum control marker used as a negative control for exosomes).

### 3.3. Proteomic Analysis of the fAd-MSCs Secretome and fAd-MSCs Exosomes

Using mass spectrometry and according to the database of peptides for Felis catus, 239 secretome proteins and 228 exosome-specific proteins were identified in fAd-MSCs. When discriminating the number of uniquely identified proteins, there were 106 secretome-specific proteins (44.3% of the total) and 95 exosome-specific proteins (41.7% of the total), with 133 proteins common between the two. The specific list of proteins, considering that a protein may be related to different functions, is shown in S1 Table.

### 3.4. Functional Annotation Based on Gene Ontology (GO)

**Figure 2:**
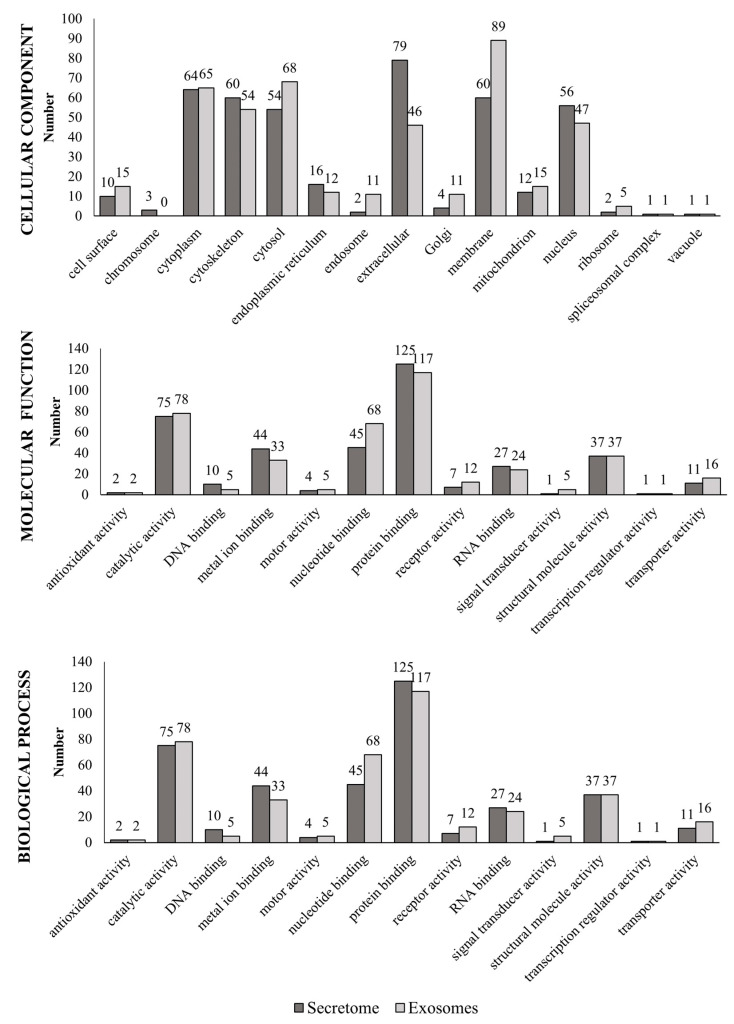
Gene Ontology (GO) annotations related to proteins found in the fAd-MSCs secretome and exosomes. Cell components, molecular functions, and biological processes of the identified proteins. A protein could be related to different parameters.

Each protein was functionally annotated (cellular component, molecular function or biological process) based on GO (Figure 2). Secretome proteins were located in the extracellular regions and in the cytoplasm, and exosomal proteins were located mainly in the membrane, the cytoplasm (referring to the entire content of a cell, excluding the plasma membrane and the nucleus but including other subcellular structures) and the cytosol (part of the cytoplasm that does not contain organelles but contains other particles, such as protein complexes).

According to function, metal ion binding was the predominant function of proteins present in the secretome, while exosomal proteins were predominantly involved in certain molecular functions, such as nucleotide-binding and transport. In addition, both the secretome and exosomes shared a series of common proteins involved in catalytic, structural, protein and RNA binding activity. The complete list of proteins is provided in Table S1.

### 3.5. Interactions between Differentially Expressed Proteins

Using STRING, a map of possible interactions among the proteins expressed differentially between the fAd-MSCs secretome and fAd-MSCs exosomes was developed. A total of 124 proteins (of the 239 total) present in the secretome were analyzed, with 377 interactions among them. Regarding the exosomes, 178 proteins (of the 228 total) were analyzed, with 370 interactions among them. Both in the secretome and in the exosomes, no significant enrichments were observed in biological pathways (reactome). The nodes with a greater number and intensity of interactions among the secretome proteins were occupied by proteins related to metabolism (procollagen-lysine, 2-oxoglutarate 5-dioxygenase 1 and aldo-keto reductase) and the PI3K-Akt signaling pathway (thrombospondin 2), among others (Figure S1 and Table S2).

Regarding exosome proteins, the most important nodes in the protein interaction network were related to metabolism (aldehyde reductase), endocytosis (heat shock proteins and members of RAS oncogene family) and pathways related to cell adhesion and cancer (catenin alpha 1 and members of the RAS oncogene family) (Figure S2 and Table S2).

### 3.6. KEGG Biological Pathway Analysis of Differentially Expressed Proteins

**Figure 3:**
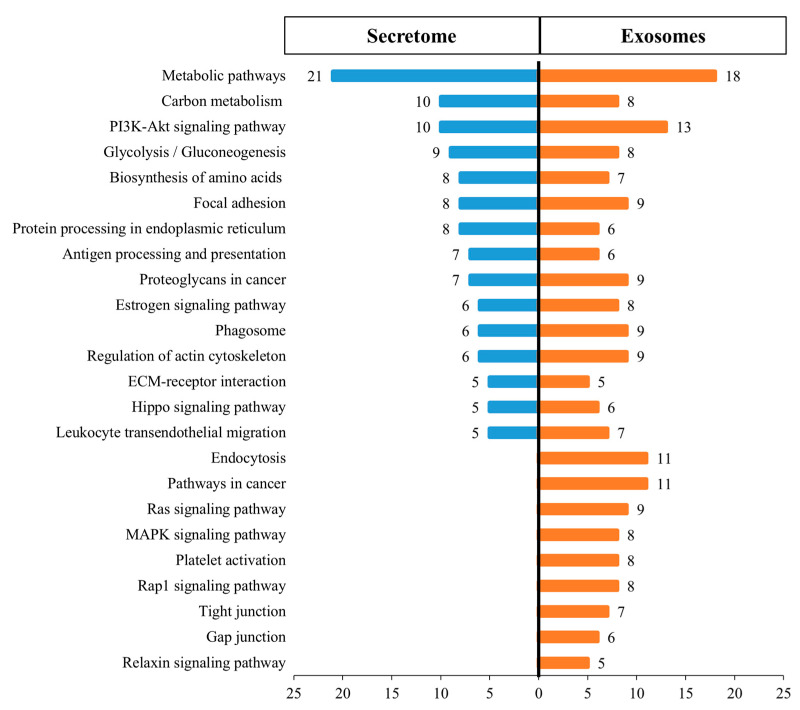
Kyoto Encyclopedia of Genes and Genomes (KEGG) pathways represented in secretome and exosome proteomes by protein numbers identified in the pathway.

**Figure 4:**
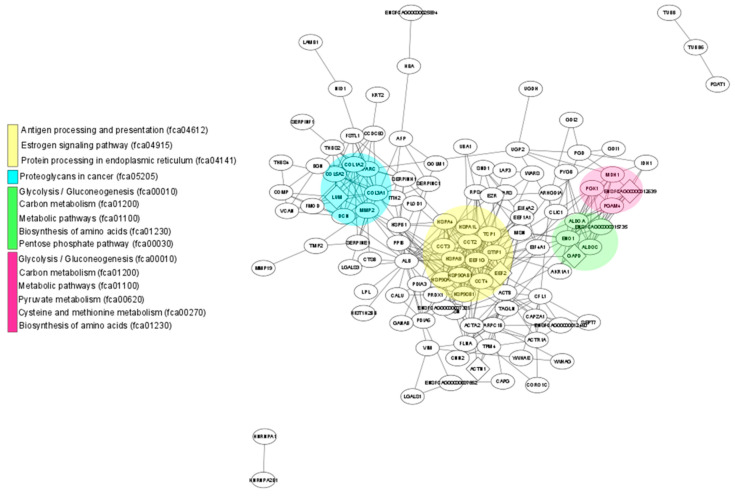
Protein network identified in the fAd-MSCs secretome. Schematic view of known and predicted interactions according to the STRING database (v.11) and visualized with Cytoscape MCODE. Each node represents a protein, and each edge represents an interaction. Only interactions with the mean confidence score (0.4) are shown. The interactions include physical and functional associations, which the evidence shows.

**Figure 5:**
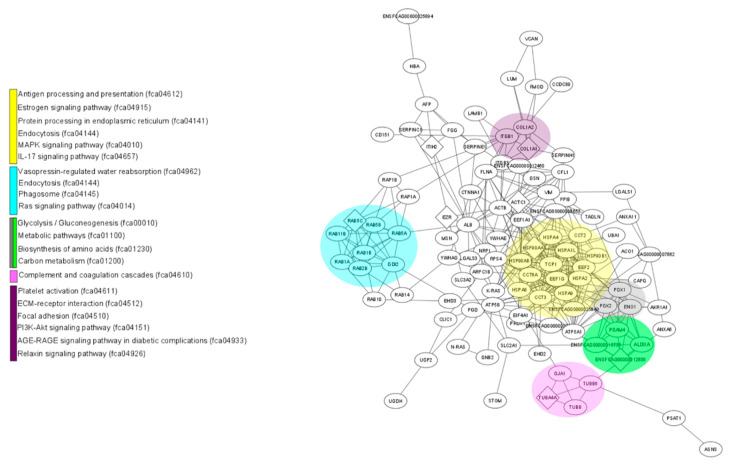
A network of proteins identified in fAd-MSCs exosomes. Schematic view of known and predicted interactions according to the STRING database (v.11) and visualized with Cytoscape MCODE. Each node represents a protein, and each edge represents an interaction. Only interactions with the mean confidence score (0.4) are shown. The interactions include physical and functional associations, which the evidence shows.

Figure 3 shows the 24 main KEGG pathways represented in the proteomes of the fAd-MSCs secretome and fAd-MSCs exosomes and the specific number of proteins identified in each case. In the secretome, proteins involved in metabolic pathways, in pathways related to the immune system and the endocrine system and in the processing of proteins in the reticulum predominated. In the exosomes, proteins involved in the processes of endocytosis, cell junctions (tight and gap junction), platelet activation and MAPK and Rap1 signaling pathways, among others, predominated. The complete list of pathways is available in S3 Table. In addition, STRING, together with visualization using Cytoscape, revealed enriched pathways associated with the KEGG database in the secretome (Figure 4) and in exosomes (Figure 5).

## 4. Discussion

The secretome and some of its components, such as exosomes, demonstrate regenerative capacities similar to the use of MSCs themselves [20,22,47]. However, some of the underlying mechanisms by which the secretome and some of its components exert their biological effects are not completely clear; therefore, considering their enormous therapeutic potential, appropriate characterization is necessary before possible clinical use.

In this study, for the first time, exosomes from fAd-MSCs were isolated; their size, morphology and expression of specific exosomal markers met the requirements of the International Society for Extracellular Vesicles (ISEV) [19,40] and were similar to those described in other domestic species and humans [17,30,31,32].

Additionally, we characterized the protein profile of the fAd-MSCs secretome and fAd-MSCs exosomes. Proteomics based on high-resolution mass spectrometry (HRMS) is a very powerful tool for protein profiling and the discovery of biomarkers in different types of biological samples, such as cell culture, tissue and biological fluid. It has been widely applied to molecular and cellular biology to elucidate biological and pathophysiological processes. In our analysis, we identified 239 proteins in the secretome and 228 proteins in the exosomes of fAd-MSCs.

After a comparative analysis and GO enrichment analysis, the proteins identified in the secretome originated in the cytoplasm or were extracellular, while the exosomal proteins originated from the membrane, cytoplasm and cytosol, coinciding with the biogenesis processes described for these MSCs components [48].

The results obtained with STRING and Cytoscape showed multiple biological interactions among the proteins identified in the secretome and the exosomes, but with some differences. The interactions among the secretome proteins were centralized in nodes related to metabolism, for example, procollagen-lysine, 2-oxoglutarate 5-dioxygenase 1 [49] (essential for the assembly and stability of collagen fibers), aldo-keto reductase [50] (enzyme that catalyzes the redox transformations involved in biosynthesis, intermediate metabolism and detoxification) and the PI3K-AKT signaling pathway [51] (crucial in numerous cellular aspects related to cell growth and survival), among others. Exosomal proteins showed a network of protein interactions related to metabolism (aldehyde reductase) [52], endocytosis, autophagy, stress response and cell death signaling (heat shock proteins) [53], and pathways related to cell adhesion (catenin alpha) [54].

These differences were confirmed by determining the main biological pathways using KEGG. In the secretome, proteins involved in metabolic pathways predominated, while in exosomes, the proteins were exclusively involved in apoptosis, endocytosis and cell junctions. There were also proteins with vasodilator, antifibrotic, and angiogenic effects (relaxin signaling pathway) [55] and linked to the complement activation and blood coagulation cascades [56]. In addition, proteins related to the MAPK, Rap1 and RAS signaling pathways were observed, i.e., GTPases that function as molecular switches for signaling pathways that regulate cell proliferation, survival, growth, migration, differentiation or dynamism of the cytoskeleton [57]; there were also proteins related to the PI3K-AKT pathway, which is involved transcription, translation, proliferation, growth and cell survival [58]. Notably, proteins were also involved in the advanced glycation end products (AGEs) signaling pathway, which is related to aging and, under certain pathological conditions, such as hyperglycemia, promotes the expression of proinflammatory cytokines such as IL-1, IL-6 and TNF-α and a variety of genes including vascular cell adhesion protein 1 (VCAM-1), tissue factor, vascular endothelial growth factor (VEGF) and advanced glycation end products (RAGE) receptor [59,60].

In addition, proteins of the secretome and exosomes shared processes mediated by the Hippo pathway and were interconnected with those in other key signaling cascades, such as those mediated by the growth factors TGF-β and Wnt [61], and proteoglycans associated with the proliferation, adhesion, angiogenesis and metastasis of cancers. Additionally, some common proteins interact with growth factors, cytokines, morphogens and enzymes [62] or intervene in the migration of leukocytes from the blood to tissue, a process that is vital for immune surveillance and inflammation [63]. Endoplasmic reticulum proteins were related to processing, folding, classification and degradation [64], and those in the phagosome were associated with tissue remodeling, inflammation and defense against infectious agents [65].

Therefore, our study identified and characterized the differences in protein components between the fAd-MSCs secretome and fAd-MSCs exosomes under standard culture conditions. This knowledge is of great importance for establishing normal levels of expression as a management strategy for the treatment of diseases as well as possible mechanisms of action [42]. In this way, deepening the understanding of the differences in protein expression between the secretome and exosomes from feline MSCs and MSCs from different origins from healthy individuals and patients affected by certain diseases can provide valuable information regarding their molecular and pathophysiological mechanisms and verify possible clinical implications, as has already been demonstrated in some diseases in humans [35]. In fact, the identification of new proteins in MSCs from sick individuals could allow faster and more reliable identification and validation of molecular markers with diagnostic value for specific conditions and of potential therapeutic targets towards which to direct new treatments.

There are some limitations in the depth of analysis due to the number of samples analyzed and the incomplete functional characterization of feline proteins in existing databases. However, our results open an opportunity for the use of the secretome and exosomes for the treatment of specific pathologies. Currently, there are four clinical studies with secretome and eleven with exosomes from MSCs where they are used as a therapeutic element [66]. In addition, numerous studies indicate that the genetic manipulation of cells or culture conditions allow the modification of the secretome and exosomes, which can enhance certain interesting biological actions for therapeutic use [67,68,69,70].

Feline MSCs largely mimic the immunomodulatory phenotype described for human, equine, and canine MSCs with some notable differences [2]. Some studies have explored the proteomic profile of different MSCs [71] and have compared the proteomics of secretomes [72]. In human MSCs, bioinformatics analysis revealed the role of exosomes in the extracellular matrix receptor. Specifically, bone marrow MSC-derived exosomes showed superior regeneration capacity, and adipose tissue MSC-derived exosomes played a significant role in immune regulation, whereas umbilical cord MSC-derived exosomes were more prominent in tissue damage repair [73]. On the other hand, we demonstrated in the canine species [17] that secretome and exosomes derived from bone marrow and adipose tissue MSCs, under standard culture conditions, contain different molecules with regenerative, pro-angiogenic, and immunomodulatory potential, whose composition depends on the tissue source, as proposed in humans [74].

New studies will improve the identification of a robust set of proteins specifically present in the MSCs secretome and exosomes, allowing further research and comparison of proteomic profiles in MSCs of different origins in the feline species.

## 5. Conclusions

This study provides a differential proteomic analysis of the fAd-MSCs secretome and fAd-MSCs exosomes to facilitate a better understanding of their mechanism of action and possible therapeutic objectives for each fraction. Our data demonstrate the existence of different proteomes, both in cellular and functional origins. This method allowed the identification of the bioactive factors that are released from fAd-MSCs and that may be responsible for the biological effects of MSCs. In addition, other molecular approaches and techniques can serve as valuable tools for the future production of cocktails of bioactive molecules or exosomes for possible therapeutic use; furthermore, these approaches and techniques can contribute to the better prediction of clinical outcomes of MSCs treatment. Additional studies that investigate differences among MSCs products are necessary to guide clinicians to choose appropriate cell products for use in specific therapeutic applications.

## Data Availability

The data presented in this study are available in this published article (and its supplementary materials).

## Supplementary Materials

The following are available online at https://www.mdpi.com/2076-2615/11/2/295/s1, Figure S1: Network of proteins identified in the fAd-MSCs secretome, Figure S2: Network of proteins identified in the fAd-MSCs exosomes, Table S1: List of specific proteins in the secretome and exosomes according to GO parameters, Table S2: Abbreviations for secretome and exosomes proteins, Table S3: Complete list of KEGG pathways in secretome and exosome proteomes.
