# Supplementary material for: Proteomic Analysis of the Secretome and Exosomes of Feline Adipose-Derived Mesenchymal Stem Cells
Source: Animals (Basel). 2021 Jan 24;11(2):295. doi: 10.3390/ani11020295 (PMC7912403; doi:10.3390/ani11020295)
Supplement: Supplementary file 1 [file animals-11-00295-s001.zip › S1 Table_List of specific proteins in the secretome and exosomes according to GO parameters.pdf]

S1 Table. List of specific proteins in fAd-MSC secretome and exosomes.

A. fAd-MSC SECRETOME									
**Number**	**Accession**	**Description**	**MW [kDa]**	**Score**	**Peptides**	**Coverage [%]**	**Biological Process**	**Cellular Component**	**Molecular Function**
1	XP_019691899.1	Basement membrane-specific heparan sulfate proteoglycan core protein isoform X2	474,4	232,602	50	15	Cell differentiation<br>Cell organization and biogenesis<br>Defense response<br>Metabolic process<br>Regulation of biological process	Extracellular	Metal ion binding<br>Protein binding
2	XP_019675142.1	Alpha-Actinin-4 isoform X5	107,3	157,467	36	43	Cell organization and biogenesis<br>Metabolic process<br>Regulation of biological process<br>Transport	Cytoskeleton	Metal ion binding<br>Protein binding
3	XP_003987818.1	Alpha-Actinin-1 isoform X4	105,4	155,021	36	44	Cell differentiation<br>Cell organization and biogenesis<br>Regulation of biological process	Cytoskeleton	Metal ion binding<br>Protein binding
4	XP_011286777.1	Tenascin isoform X5	231,1	137,093	31	23	Cell communication<br>Cell differentiation<br>Cell organization and biogenesis<br>Metabolic process<br>Regulation of biological process<br>Response to stimulus	Extracellular	Protein binding
5	XP_019687667.1	Poly [ADP-ribose] polymerase 6 isoform X2	126,1	110,614	19	16	Metabolic process<br>Regulation of biological process	Nucleus	Enzyme regulator activity<br>Nucleotide binding<br>Protein binding
6	XP_011284015.1	Collagen alpha-2(VI) chain isoform X1	108,7	84,769	19	19	Cell differentiation<br>Cell organization and biogenesis<br>Metabolic process<br>Regulation of biological process<br>Response to stimulus<br>Transport	Cytoplasm<br>Extracellular	Protein binding<br>Structural molecule activity
7	XP_003993920.1	Actin, aortic smooth muscle	42	82,572	15	49	Regulation of biological process	Cytoplasm	Nucleotide binding
8	XP_003991010.1	Collagen alpha-1(III) chain	138,6	73,559	17	13	Cell differentiation<br>Cell organization and biogenesis<br>Metabolic process<br>Regulation of biological process<br>Response to stimulus<br>Transport	Cytoplasm<br>Extracellular	Protein binding<br>Structural molecule activity

9	XP_003998091.1	72 kDa type IV Collagenase	65,6	68,269	18	28	Metabolic process	Cytoplasm<br>Extracellular	Catalytic activity<br>Metal ion binding
10	XP_011287018.1	Collagen alpha-1(V) chain	183,6	64,56	11	8	Cell differentiation<br>Cell organization and biogenesis<br>Metabolic process<br>Regulation of biological process<br>Response to stimulus<br>Transport	Cytoplasm<br>Extracellular	Structural molecule activity
11	XP_004001049.2	Biglycan	41,9	58,052	9	30	Metabolic process<br>Regulation of biological process<br>Response to stimulus	Cell surface<br>Cytoplasm<br>Extracellular<br>Membrane	Enzyme regulator activity<br>Protein binding
12	XP_011283223.1	Periostin isoform X1	93	57,592	15	24	Cell communication<br>Cell organization and biogenesis<br>Regulation of biological process<br>Response to stimulus	Extracellular	Protein binding
13	XP_019679760.1	Plastin-3 isoform X1	71,7	56,491	15	29	Cell organization and biogenesis	Cytoskeleton<br>Cytosol<br>Membrane	Metal ion binding<br>Protein binding
14	XP_003987270.1	Protein disulfide-isomerase A3	56,8	54,085	16	36	Cellular homeostasis<br>Metabolic process<br>Regulation of biological process<br>Response to stimulus	Cell surface<br>Endoplasmic reticulum<br>Nucleus	Catalytic activity<br>Protein binding<br>RNA binding
15	XP_003989548.1	ProCollagen-lysine,2-oxoglutarate 5-dioxygenase 1	83,4	53,261	12	23	Metabolic process<br>Response to stimulus	Endoplasmic reticulum	Catalytic activity<br>Metal ion binding
16	XP_003993948.1	Nidogen-1	135,7	46,036	13	13	Cell organization and biogenesis<br>Regulation of biological process	Cell surface<br>Membrane	Metal ion binding<br>Protein binding
17	XP_003996465.1	Pigment epithelium-derived factor	46,2	39,831	10	31	Regulation of biological process	Extracellular	Enzyme regulator activity
18	XP_006942048.3	ProCollagen C-endopeptidase enhancer 1 isoform X1	59,5	38,883	9	23	Metabolic process<br>Regulation of biological process	Cytoplasm<br>Extracellular	Protein binding<br>Structural molecule activity
19	XP_003996537.1	Fructose-bisphosphate aldolase C	39,3	38,034	5	19	Cell differentiation<br>Metabolic process	Mitochondrion	Catalytic activity<br>Protein binding
20	XP_019680743.1	Adipocyte enhancer-binding protein 1 isoform X1	129,6	37,741	11	12	Metabolic process	Extracellular<br>Nucleus	DNA binding<br>Metal ion binding<br>Protein binding
21	XP_003991011.1	Collagen alpha-2(V) chain	145,1	37,698	12	10	Cell organization and biogenesis<br>Regulation of biological process<br>Response to stimulus	Cytoplasm<br>Extracellular	Protein binding<br>Structural molecule activity
22	XP_006935150.2	Extracellular matrix protein 1 isoform X1	63,4	34,958	11	23	Regulation of biological process<br>Response to stimulus	Extracellular	Protein binding

23	XP_004000294.2	Matrix-remodeling-associated protein 5	310,8	34,166	18	8	Metabolic process	Extracellular	Protein binding
24	XP_019671105.1	Pappalysin-1	169,9	33,472	7	6	Metabolic process<br>Regulation of biological process	Extracellular	Catalytic activity<br>Metal ion binding
25	XP_003999362.2	Sulfhydryl oxidase 1	80,3	33,134	10	16	Cellular homeostasis<br>Metabolic process<br>Regulation of biological process	Extracellular	Catalytic activity
26	XP_019671735.1	Tropomyosin beta chain isoform X3	33	32,771	9	19	Cell differentiation<br>Cell organization and biogenesis	Cytoplasm<br>Cytosol	Protein binding
27	XP_006933529.1	Complement C1s subcomponent	77,7	31,863	9	14	Metabolic process	Extracellular	Catalytic activity<br>Metal ion binding
28	XP_003981423.1	SPARC	34,5	31,335	5	11	Regulation of biological process<br>Response to stimulus	Nucleus<br>Extracellular	Metal ion binding<br>Protein binding
29	XP_006932020.3	Laminin subunit alpha-4 isoform X1	211,4	30,808	11	7	Cell differentiation<br>Cell organization and biogenesis<br>Cellular component movement<br>Metabolic process<br>Regulation of biological process<br>Transport	Extracellular	Catalytic activity<br>Motor activity<br>Nucleotide binding<br>Protein binding<br>Structural molecule activity<br>Transporter activity
30	XP_006941079.1	Nucleobindin-1 isoform X1	53,8	29,324	11	26	Regulation of biological process	Nucleus	Metal ion binding
31	XP_019673334.1	Puromycin-sensitive aminopeptidase isoform X1	94,1	27,694	11	16	Metabolic process	Cytosol<br>Nucleus	Metal ion binding<br>Protein binding
32	XP_019690013.1	Complement C1r subcomponent isoform X1	81,5	27,628	6	9	Metabolic process	Extracellular	Catalytic activity<br>Metal ion binding
33	XP_011288149.1	Apolipoprotein E	30,2	26,983	6	29	Metabolic process<br>Transport	Extracellular	Protein binding<br>Transporter activity
34	NP_001009307.1	Glyceraldehyde-3-phosphate dehydrogenase	35,8	25,421	7	28	Cell death<br>Cell organization and biogenesis<br>Metabolic process<br>Regulation of biological process	Cytoplasm<br>Cytoskeleton<br>Cytosol<br>Nucleus	Catalytic activity<br>Nucleotide binding<br>Protein binding
35	XP_019678430.1	Transketolase isoform X1	68,5	24,138	7	17	metabolic process	Nucleus	Catalytic activity
36	XP_011290023.2	Metalloproteinase inhibitor 1, partial	19,7	22,844	5	37	Regulation of biological process<br>Response to stimulus	Extracellular	Metal ion binding<br>Protein binding
37	XP_003989159.1	Decorin	39,9	20,293	6	19	Metabolic process<br>Regulation of biological process<br>Response to stimulus	Cytoplasm<br>Extracellular	Enzyme regulator activity<br>Protein binding<br>RNA binding
38	XP_011279272.1	Growth arrest-specific protein 6 isoform X1	70,9	19,844	6	14	Cell growth	Cell Surface<br>Extracellular<br>Membrane	Metal ion binding
39	XP_019666653.1	Target of Nesh-SH3	121,5	19,822	4	5	Cell organization and biogenesis<br>Regulation of biological process	Extracellular	Protein binding

40	XP_019674453.1	Tropomyosin alpha-4 chain isoform X3	28,1	19,245	7	22	Cell differentiation<br>Cell organization and biogenesis	Cytoplasm<br>Cytosol	Protein binding
41	XP_019667649.1	Spondin-1, partial	93,8	19,152	6	12	Development	Extracellular	Protein binding
42	NP_001036032.1	Lipoprotein lipase precursor	53,6	18,669	5	14	Metabolic process<br>Response to stimulus	Extracellular<br>Membrane	Catalytic activity<br>Protein binding
43	XP_003982066.1	Tropomyosin alpha-4 chain isoform X1	32,7	17,319	7	20	Cell differentiation<br>Cell organization and biogenesis	Cytoplasm<br>Cytosol	Protein binding
44	XP_019678084.1	Tropomyosin alpha-3 chain isoform X9	29,4	17,158	7	23	Cell differentiation<br>Cell organization and biogenesis	Cytoplasm<br>Cytosol	Protein binding
45	XP_019681388.1	Caldesmon isoform X3	62,7	16,981	7	13	Response to stimulus	Cytoskeleton	Protein binding
46	XP_019671386.1	Golgi membrane protein 1	45,4	16,676	6	23	Cell organization and biogenesis<br>Regulation of biological process	Golgi	Receptor activity
47	XP_019673902.1	Galectin-3-binding protein isoform X1	107,2	16,386	4	5	Cell differentiation<br>Defense response<br>Metabolic process<br>Response to stimulus	Nucleus<br>Cytoplasm<br>Mitochondrion<br>Membrane<br>Extracellular	Protein binding
48	XP_003982756.1	Semaphorin-3C	85,1	16,163	9	14	Cellular component movement<br>Regulation of biological process<br>Response to stimulus	Membrane	Protein binding
49	XP_019687862.1	Tropomyosin alpha-1 chain isoform X4	28,7	15,622	5	16	Cell differentiation<br>Cell organization and biogenesis	Cytoplasm<br>Cytosol	Protein binding
50	XP_003997297.1	Metalloproteinase inhibitor 2	24,5	15,086	3	10	Regulation of biological process<br>Response to stimulus	Extracellular	Enzyme regulator activity<br>Metal ion binding<br>Protein binding
51	XP_011278837.1	Staphylococcal nuclease domain-containing protein 1	101,9	14,509	5	8	Cell differentiation<br>Metabolic process<br>Regulation of biological process	Cytoplasm<br>Cytosol<br>Membrane<br>Mitochondrion<br>Nucleus	Catalytic activity<br>Protein binding<br>RNA binding
52	XP_011281387.1	Gremlin-1	20,7	14,118	3	28	Cell communication<br>Cell organization and biogenesis<br>Cellular component movement<br>Regulation of biological process<br>Response to stimulus	Cell Surface<br>Extracellular	Enzyme regulator activity<br>Protein binding
53	XP_003988911.1	Matrix metalloproteinase-19	61,5	14,058	4	13	Metabolic process	Extracellular	Catalytic activity<br>Metal ion binding
54	XP_019667038.1	Complement component C7 isoform X1	95,3	14,049	3	5	Metabolic process<br>Regulation of biological process<br>Response to stimulus	Extracellular	Catalytic activity<br>Metal ion binding
55	XP_019684940.1	Afamin	68,7	13,873	4	8	Regulation of biological process<br>Response to stimulus	Extracellular	Metal ion binding

							Transport		
56	XP_006935577.1	Isocitrate dehydrogenase [NADP] cytoplasmic	46,7	13,729	7	19	Metabolic process	Cytoplasm<br>Cytosol<br>Mitochondrion	Catalytic activity<br>Metal ion binding<br>Nucleotide binding
57	XP_011288783.1	ProCollagen-lysine,2-oxoglutarate 5-dioxygenase 3	85,7	13,677	3	6	Metabolic process	Cytoplasm<br>Extracellular	Catalytic activity
58	XP_003985758.1	Histone H1.3	22,1	13,607	5	15	Cell organization and biogenesis<br>Metabolic process<br>Regulation of biological process	Chromosome<br>Nucleus	DNA binding<br>RNA binding
59	XP_011289480.1	Thrombospondin-3	103,9	13,528	5	8	Regulation of biological process	Extracellular	Metal ion binding
60	XP_011280382.1	Histone H2B type 1-like	20	13,334	4	19	Cell organization and biogenesis<br>Regulation of biological process	Chromosome<br>Nucleus	DNA binding
61	XP_003984894.1	Cathepsin B isoform X1	37,7	13,102	4	17	Cell differentiation<br>Metabolic process<br>Regulation of biological process<br>Response to stimulus	Mitochondrion<br>Vacuole	Catalytic activity<br>Protein binding
62	XP_006932208.1	Thrombospondin-2 isoform X1	129,5	12,783	6	6	Regulation of biological process	Extracellular	Metal ion binding<br>Protein binding
63	NP_001009329.1	Malate dehydrogenase, cytoplasmic	36,4	12,639	5	20	metabolic process	Cytoplasm<br>Cytosol<br>Mitochondrion	Catalytic activity
64	XP_003994413.1	Alpha-centrActin	42,6	12,569	3	13	Cell organization and biogenesis<br>Cellular component movement<br>Regulation of biological process	Cytoplasm<br>Cytoskeleton	Nucleotide binding
65	XP_019683254.1	Latent-transforming growth factor beta-binding protein 1 isoform X1	171,7	11,979	5	5	Cell death<br>Cell differentiation<br>Cell growth<br>Cell organization and biogenesis<br>Cell proliferation<br>Cellular component movement<br>Metabolic process<br>Regulation of biological process<br>Response to stimulus<br>Transport	Extracellular	Metal ion binding<br>Receptor activity
66	XP_011278043.1	Keratin, type II cytoskeletal 6A, partial	49,5	11,821	4	8	Metabolic process	Cytoskeleton<br>Membrane<br>Nucleus	Protein binding<br>structural molecule activity
67	AEP60131.1	Beta-N-acetylhexosaminidase beta subunit	61,2	11,81	3	7	Metabolic process	Endosome	Catalytic activity
68	XP_019666613.1	Collagen alpha-1(XVIII) chain	172,8	11,291	5	4	Cell organization and biogenesis<br>Regulation of biological process<br>Response to stimulus	Cytoplasm<br>Extracellular	Protein binding<br>Structural molecule activity

69	XP_003982994.1	Septin-7 isoform X1	50,6	11,285	4	13	Regulation of biological process<br>Response to stimulus	Cytoskeleton<br>Membrane	Nucleotide binding<br>Protein binding
70	XP_003982134.1	Cartilage oligomeric matrix protein	82,3	11,236	4	7	Regulation of biological process	Extracellular	Metal ion binding<br>Protein binding
71	XP_019687157.1	CD109 antigen	164,2	11,125	5	4	Regulation of biological process	Membrane	Protein binding
72	XP_003983065.1	Calumenin isoform X1	37,1	11,034	4	11	Metabolic process<br>Regulation of biological process<br>Response to stimulus	Endoplasmic reticulum	Metal ion binding
73	XP_006935306.2	Actin-related protein 3	47,7	10,883	5	15	Cell organization and biogenesis<br>Cellular component movement<br>Regulation of biological process	Cytoskeleton	Protein binding<br>Structural molecule activity
74	XP_003997322.1	Rho GDP-dissociation inhibitor 1	23,3	10,757	3	21	Regulation of biological process	Cytoplasm	Enzyme regulator activity
75	XP_006937245.1	Reticulocalbin-1, partial	31	10,634	4	20	Cell differentiation<br>Cell organization and biogenesis<br>Regulation of biological process<br>Response to stimulus	Endoplasmic reticulum	Metal ion binding
76	XP_003991859.1	Eukaryotic initiation factor 4A-II	46,4	9,862	4	14	Metabolic process<br>Regulation of biological process	Cytoplasm	Catalytic activity<br>DNA binding<br>Nucleotide binding<br>RNA binding
77	XP_011283864.1	Actin-related protein 2/3 complex subunit 2	34,3	9,562	3	12	Cell organization and biogenesis<br>Regulation of biological process	Cytoskeleton	Protein binding<br>Structural molecule activity
78	XP_019690700.1	DynActin subunit 2 isoform X1	44,9	9,359	3	11	Cell organization and biogenesis<br>Cellular component movement<br>Regulation of biological process	Cytoskeleton<br>Cytoplasmic	Transporter activity
79	XP_003988191.1	Plexin domain-containing protein 2 isoform X1	59,5	9,282	3	6	Development<br>Metabolic process<br>Regulation of biological process	Membrane	Receptor activity
80	XP_003990521.1	F-Actin-capping protein subunit alpha-1 isoform X1	33	I78:J78	3	15	Cell organization and biogenesis<br>Regulation of biological process	Cytoskeleton	Protein binding<br>Structural molecule activity
81	XP_019666549.1	Programmed cell death 6-interActing protein isoform X1	95,1	8,769	3	5	Cell death<br>Cell organization and biogenesis<br>Metabolic process<br>Regulation of biological process<br>Response to stimulus<br>Transport	Cytoplasm<br>Cytosol	Protein binding
82	XP_003993602.1	Stress-induced-phosphoprotein 1	62,5	8,718	3	6	Metabolic process	Nucleus	Protein binding
83	XP_003985774.1	Histone H2B type 1-B	13,9	8,555	3	26	Cell organization and biogenesis	Chromosome<br>Nucleus	DNA binding<br>Protein binding
84	XP_003981650.1	Calponin-2 isoform X1	33,2	8,536	3	12	Cell organization and biogenesis<br>Regulation of biological process<br>Response to stimulus	Cytoskeleton	Protein binding

85	XP_003981157.1	Thrombospondin-4	105,9	8,5	3	4	Regulation of biological process	Extracellular	Metal ion binding
86	XP_003984083.1	T-complex protein 1 subunit delta	58	8,38	4	8	Cell organization and biogenesis<br>Metabolic process<br>Regulation of biological process<br>Transport	Cytoskeleton<br>Cytosol	Nucleotide binding
87	XP_006930072.1	Glycogen phosphorylase, brain form	96,5	8,318	3	4	Metabolic process	Cytoplasm<br>Membrane	Catalytic activity
88	XP_003998956.1	Vasorin	72,2	8,26	3	5	Regulation of biological process<br>Response to stimulus	Cell Surface<br>Membrane<br>Mitochondrion	Protein binding
89	XP_004001071.1	Rab GDP dissociation inhibitor alpha	50,5	8,056	4	11	Metabolic process<br>Regulation of biological process<br>Response to stimulus<br>Transport	Cytoplasm	Catalytic activity<br>Enzyme regulator activity
90	XP_003993564.1	Neutral alpha-glucosidase AB isoform X1	109	7,989	4	7	Metabolic process	Membrane	Catalytic activity<br>RNA binding
91	XP_003981521.1	Threonine--tRNA ligase, cytoplasmic	83,5	7,959	4	6	Metabolic process	Cytoplasm<br>Cytoskeleton<br>Cytosol	Catalytic activity<br>Nucleotide binding
92	XP_003998589.1	Heat shock protein beta-1	22,7	7,879	3	18	Metabolic process<br>Regulation of biological process<br>Response to stimulus	Cytoplasm<br>Membrane<br>Nucleus	Enzyme regulator activity<br>Protein binding<br>RNA binding
93	XP_003984581.1	Protein disulfide-isomerase A6	48,4	7,629	3	9	Cellular homeostasis<br>Regulation of biological process	Cell Surface<br>Endoplasmic reticulum<br>Nucleus	Catalytic activity<br>Protein binding<br>RNA binding
94	XP_011289248.1	Aminopeptidase B	73,4	7,5	3	7	Metabolic process	Membrane	Catalytic activity<br>Metal ion binding
95	XP_019680058.1	Glucose-6-phosphate 1-dehydrogenase isoform X1	63,7	7,402	3	5	Metabolic process	Cytoplasm	Catalytic activity
96	XP_019670205.1	Coronin-1C	53,2	6,189	3	6	Cell organization and biogenesis<br>Cellular component movement<br>Regulation of biological process	Cytoskeleton	Protein binding
97	XP_006932077.1	Connective tissue growth factor	42,8	6,145	3	9	Cell growth	Extracellular	Protein binding
98	XP_003991726.1	Follistatin-related protein 1	34,7	6,086	3	9	Response to stimulus<br>Transport	Membrane	Metal ion binding<br>Protein binding<br>Transporter activity
99	XP_006936266.1	Alpha-2-HS-glycoprotein	38,7	5,688	3	6	Defense response<br>Regulation of biological process<br>Response to stimulus	Extracellular	Enzyme regulator activity
100	XP_011286169.1	Putative phospholipase B-like 2, partial	62,7	5,443	3	5	Metabolic process	Extracellular	Catalytic activity
101	XP_003985554.1	Cytosol aminopeptidase	56,9	5,095	3	7	Metabolic process	Cytosol	Catalytic activity

102	XP_011285435.1	Coronin-1B	53,9	4,93	3	6	Cell organization and biogenesis<br>Cellular component movement<br>Regulation of biological process	Cytoskeleton	Protein binding
103	XP_003988842.1	Heterogeneous nuclear ribonucleoprotein A1 isoform X1	38,8	4,865	3	11	Metabolic process<br>Regulation of biological process<br>Transport	Nucleus	RNA binding
104	XP_019695128.1	Vitamin K-dependent protein S isoform X1	75,4	4,654	3	4	Transport	Endoplasmic reticulum<br>Extracellular<br>Golgi	Catalytic activity
105	XP_003991983.1	Neuroserpin	46,4	4,596	3	8	regulation of biological process	Organelle lumen	Enzyme regulator activity
106	XP_019673866.1	Septin-9 isoform X1	68,7	4,504	3	5	Regulation of biological process<br>Response to stimulus	Cytoskeleton<br>Membrane	Nucleotide binding<br>Protein binding

B. fAd-MSC EXOSOMES									
Number	Accession	Description	MW [kDa]	Score	Peptides	Coverage [%]	Biological Process	Cellular Component	Molecular Function
1	XP_019691898.1	Basement Membrane-Specific Heparan Sulfate Proteoglycan Core Protein Isoform X1	475	84,2	31	8	Regulation of biological process<br>Development<br>Coagulation	Extracellular	Protein binding
2	XP_003987346.1	Actin, Alpha Cardiac Muscle 1	42	74,054	14	45	Cell organization and biogenesis<br>Cellular component movement<br>Regulation of biological process	Extracellular	Protein binding
3	XP_011283389.1	Prostaglandin F2 Receptor Negative Regulator	98,4	52,603	17	20	Cell organization and biogenesis	Extracellular	Protein binding
4	XP_003981430.1	Annexin A6 Isoform X2	75,3	47,475	18	31	Cell organization and biogenesis<br>Metabolic process<br>Regulation of biological process<br>Coagulation<br>Transport	Cytoplasm<br>Cytosol	Metal ion binding
5	XP_011287322.1	Unconventional Myosin-Ic Isoform X2	119,7	46,555	15	16	Cell differentiation<br>Cell organization and biogenesis<br>Cellular component movement<br>Metabolic process<br>Regulation of biological process<br>Transport	Cytoskeleton	Catalytic activity<br>Motor activity<br>Nucleotide binding
6	XP_019689386.1	Cytoplasmic Dynein 1 Heavy Chain 1	523,4	40,486	24	6	Regulation of biological process<br>Transport	Cytoplasm<br>Cytosol	Motor activity
7	XP_003996748.1	Collagen Alpha-1(I) Chain Isoform X1	138,8	40,071	14	13	Cell differentiation<br>Cell organization and biogenesis<br>Metabolic process<br>Regulation of biological process<br>Response to stimulus<br>Transport	Cytoplasm<br>Extracellular	Protein binding<br>Structural molecule activity
8	XP_011283388.1	Sodium/Potassium-Transporting AtPase Subunit Alpha-1	112,7	38,411	12	14	Cellular homeostasis<br>metabolic Process<br>Regulation of biological process<br>Response to stimulus<br>Transport	Mitochondrion	Metal ion binding<br>Nucleotide binding
9	XP_011288171.1	Alpha-Actinin-4 Isoform X1	104,9	38,29	16	17	Cell organization and biogenesis<br>Cellular component movement<br>Regulation of biological process<br>Response to stimulus<br>Transport	Extracellular	Metal ion binding<br>Protein binding
10	XP_006939152.1	Annexin A1 Isoform X1	38,6	35,022	7	28	Coagulation<br>Transport<br>Cell organization and biogenesis<br>Metabolic process<br>Regulation of biological process	Extracellular	Metal ion binding
11	XP_003993621.2	EH Domain-Containing Protein 1	62	34,765	10	23	Cell organization and biogenesis<br>Regulation of biological process<br>Transport	Cytoplasm<br>Cytosol	Nucleotide binding<br>Protein binding

12	XP_006932972.1	Heat Shock-Related 70 Kda Protein 2	70	34,053	8	15	Cell organization and biogenesis<br>Response to stimulus	Cytoplasm<br>Cytosol	Nucleotide binding
13	XP_019688751.1	Alpha-Actinin-1 Isoform X1	109	33,599	9	11	Cell differentiation<br>Cell organization and biogenesis<br>Regulation of biological process	Extracellular	Metal ion binding<br>Protein binding
14	XP_011285729.1	Myoferlin Isoform X1	236,5	33,257	14	8	Cell organization and biogenesis<br>Regulation of biological process<br>Response to stimulus	Membrane	Protein binding
15	XP_019666550.1	Programmed Cell Death 6-Interacting Protein Isoform X2	94,4	33,034	11	15	Cell death<br>Cell organization and biogenesis<br>Regulation of biological process	Cytoplasm<br>Cytosol	Protein binding
16	XP_003999437.1	Plasma Membrane Calcium-Transporting Atpase 4 Isoform X7	137,9	32,626	14	13	Cellular component movement<br>Cellular homeostasis<br>Regulation of biological process<br>Response to stimulus<br>Transport	Membrane	Catalytic activity<br>Metal ion binding<br>Nucleotide binding<br>Transporter activity
17	XP_003988964.1	ATP Synthase Subunit Beta, Mitochondrial	56,4	25,537	8	21	Metabolic process<br>Transport	Mitochondrion	Catalytic activity<br>Nucleotide binding<br>Transporter activity
18	XP_003991220.1	Tubulin Alpha-4A Chain	49,9	24,588	7	19	Cell organization and biogenesis<br>Response to stimulus	Cytoplasm<br>Cytoskeleton<br>Nucleus	Catalytic activity<br>Nucleotide binding<br>Structural molecule activity
19	P11979.2	Pyruvate Kinase PKM	58	22,535	12	23	Metabolic process<br>Response to stimulus	Cytoplasm<br>Nucleus	Catalytic activity<br>Metal ion binding<br>Nucleotide binding
20	XP_006936504.1	Raftlin	61,2	21,577	7	16	Cell organization and biogenesis<br>Regulation of biological process<br>Response to stimulus<br>Transport	Cytoplasm<br>Endosome<br>membrane	RNA binding
21	XP_006933500.1	Glyceraldehyde-3-Phosphate Dehydrogenase Isoform X1	35,8	21,53	3	12	Metabolic process	Cytoplasm<br>Cytosol	Catalytic activity<br>Nucleotide binding
22	XP_006944006.1	Plastin-3 Isoform X2	70,7	19,652	7	13	Cell organization and biogenesis	Cytoskeleton<br>Cytosol<br>Membrane	Metal ion binding<br>Protein binding
23	XP_003995863.1	Erythrocyte Band 7 Integral Membrane Protein	31,3	19,481	7	30	Cell organization and biogenesis<br>Regulation of biological process	Cytoplasm<br>Cytoskeleton<br>Cytosol<br>Endoplasmic reticulum<br>Membrane<br>Mitochondrion	Protein binding
24	XP_003994143.1	Annexin A11	54,4	19,02	8	18	Response to stimulus<br>Transport	Cytoplasm<br>Cytosol<br>Membrane<br>vacuole	Metal ion binding<br>Protein binding<br>RNA binding
25	XP_011280428.1	Flotillin-1	47,3	18,75	8	21	Cell organization and biogenesis<br>Regulation of biological process<br>Response to stimulus<br>Transport	Cytoplasm	Signal transducer activity

26	XP_011281051.1	Lactadherin Isoform X1	47,9	16,818	8	18	Cell growth<br>Cell organization and biogenesis<br>Metabolic process<br>Regulation of metabolic process	Extracellular	Protein binding
27	XP_011282254.1	Keratin, Type II Cytoskeletal 6A	60,8	15,267	6	6	Transport	Cytoskeleton<br>Membrane<br>Nucleus	Structural molecule activity<br>Transporter activity
28	XP_003986563.3	Gap Junction Alpha-1 Protein	43,1	14,511	3	14	Cell communication<br>Regulation of biological process<br>Response to stimulus<br>Transport	Membrane<br>Mitochondrion	Signal transducer activity<br>Transporter activity
29	XP_003989073.1	Ras-Related Protein Rap-1B	20,9	14,308	6	27	Cell proliferation<br>Regulation of biological process<br>Response to stimulus	Cytosol<br>Membrane	Catalytic activity<br>Nucleotide binding<br>Protein binding
30	XP_003996927.1	Ras-Related Protein Rab-5C	23,5	14,18	5	27	Regulation of biological process<br>Response to stimulus<br>Transport	Endosome<br>Membrane	Catalytic activity<br>Nucleotide binding
31	XP_003996910.3	Keratin, Type I Cytoskeletal 16	51,8	13,898	7	10	Transport	Cytoskeleton<br>Membrane<br>Nucleus	Structural molecule activity<br>Transporter activity
32	XP_019693915.1	Integrin Alpha-V	108,9	13,625	5	6	Cell differentiation<br>Cell growth<br>Cell organization and biogenesis<br>Cellular component movement<br>Metabolic process<br>Regulation of biological process<br>Response to stimulus<br>Transport	Cell surface<br>Endosome<br>Membrane	Metal ion binding<br>Protein binding<br>Receptor activity
33	XP_003997581.1	60S Ribosomal Protein L18	21,6	13,222	4	26	Metabolic process	Ribosome	Structural molecule activity
34	XP_006935061.1	Ras-Related Protein Rap-1A Isoform X1	21	12,901	6	27	Regulation of biological process<br>Response to stimulus<br>Transport	Cytoplasm<br>Membrane	Catalytic activity<br>Nucleotide binding<br>Protein binding<br>Transporter activity
35	XP_006939383.2	Tropomyosin Beta Chain Isoform X1	32,8	12,702	5	12	Cell organization and biogenesis<br>Regulation of biological process<br>Response to stimulus	Cytoplasm<br>Cytosol	Protein binding
36	XP_019690541.1	Keratin, Type Ii Cytoskeletal 3	63	12,213	5	6	Cell differentiation<br>Metabolic process	Cytoskeleton<br>Membrane<br>Nucleus	Structural molecule activity<br>Transporter activity
37	NP_001041625.1	Integrin Beta-1 Precursor	88	11,639	5	6	Metabolic process<br>Regulation of biological process<br>Response to stimulus<br>Transport	Cell surface<br>Endosome<br>Membrane	Metal ion binding<br>Protein binding<br>Receptor activity
38	XP_003993854.1	CD151 Antigen	28,1	11,177	3	13	Cell proliferation<br>Cellular component movement<br>Regulation of biological process<br>Response to stimulus	Membrane	Protein binding

39	XP_011280661.1	Catenin Alpha-1	100	11,164	5	6	Cell organization and biogenesis<br>Regulation of biological process<br>Response to stimulus	Cytoskeleton<br>Cytosol<br>Golgi<br>Membrane	Protein binding<br>RNA binding<br>Structural molecule activity
40	XP_003990014.1	Solute Carrier Family 2, Facilitated Glucose Transporter Member 1	54	11,125	5	13	Cell communication<br>Cell organization and biogenesis<br>Response to stimulus<br>Transport	Cytoplasm<br>Cytoskeleton<br>Cytosol<br>Membrane<br>Nucleus	Protein binding<br>Transporter activity
41	XP_019694789.1	Transferrin Receptor Protein 1 Isoform X1	112,7	10,939	4	4	Cellular homeostasis<br>Transport	Cytosol<br>Membrane	Catalytic activity<br>Protein binding<br>Signal transducer activity
42	XP_011281682.1	Glycogen Phosphorylase, Liver Form	111,3	10,87	4	4	Metabolic process	Cytoplasm	Catalytic activity
43	XP_011279416.1	Annexin A4	35,9	10,865	5	17	Cell differentiation<br>Regulation of biological process<br>Response to stimulus	Cell surface<br>Cytoplasm<br>Membrane<br>Nucleus	Metal ion binding<br>Protein binding
44	XP_003988849.2	Integrin Alpha-5	119,7	10,821	4	5	Cell differentiation<br>Cell organization and biogenesis<br>Cellular component movement<br>Regulation of biological process<br>Response to stimulus	Cell surface<br>Endosome<br>Membrane	Metal ion binding<br>Protein binding<br>Receptor activity
45	XP_003984399.1	EH Domain-Containing Protein 3	60,9	10,559	3	6	Cell organization and biogenesis<br>Metabolic process<br>Regulation of biological process<br>Transport	Cytoplasm<br>Cytosol	Nucleotide binding<br>Protein binding
46	XP_003981831.1	Ras-Related Protein Rab-11B	24,5	10,512	4	21	Regulation of biological process<br>Response to stimulus	Cytoplasm<br>Cytosol	Protein binding
47	XP_003997662.1	EH Domain-Containing Protein 2	61,3	10,352	4	7	Cell organization and biogenesis<br>Regulation of biological process<br>Transport	Cytoplasm<br>Cytosol<br>Membrane	Metal ion binding<br>Nucleotide binding<br>Protein binding
48	XP_011287294.1	Flotillin-2 Isoform X1	47	10,225	4	10	Cell organization and biogenesis<br>Regulation of biological process	Extracellular	Metal ion binding
49	XP_019689425.1	Integrin Beta-1 Isoform X1	88,6	10,033	5	5	Cell differentiation<br>Cell growth<br>Cell organization and biogenesis<br>Cellular component movement<br>Metabolic process<br>Regulation of biological process<br>Response to stimulus<br>Transport	Cell surface<br>Endosome<br>Membrane	Metal ion binding<br>Protein binding<br>Receptor activity
50	XP_003998542.1	Guanine Nucleotide-Binding Protein G(I)/G(S)/G(T) Subunit Beta-2	37,3	9,808	3	9	Regulation of biological process<br>Response to stimulus	Membrane	Protein binding
51	XP_011282716.1	EGF-Like Repeat And Discoidin I-Like Domain-Containing Protein 3 Isoform X1	54	9,746	4	9	Cell organization and biogenesis<br>Regulation of biological process	Extracellular	Protein binding

52	XP_019693224.1	GTPase NRas	21,2	9,617	3	19	Regulation of biological process<br>Response to stimulus	Golgi<br>Membrane	Catalytic activity<br>Nucleotide binding<br>Protein binding
53	XP_003997084.1	Integrin Beta-3 Isoform X2	86,6	9,4	5	7	Cell differentiation<br>Cell growth<br>Cell organization and biogenesis<br>Cellular component movement<br>Metabolic process<br>Regulation of biological process<br>Response to stimulus<br>Transport	Cell Surface<br>Endosome<br>Membrane	Metal ion binding<br>Protein binding<br>Receptor activity
54	XP_019667819.1	CD44 Antigen Isoform X1	81,6	9,323	4	5	Cellular component movement<br>Metabolic process<br>Regulation of biological process<br>Response to stimulus	Cell Surface	Protein binding
55	XP_003986275.1	Phosphoglycerate Kinase 2	44,8	9,254	4	12	Cellular component movement<br>Metabolic process	Nucleus	Catalytic activity<br>Nucleotide binding
56	XP_003988919.1	Ras-Related Protein Rab-5B	23,7	9,083	4	21	Regulation of biological process<br>Response to stimulus	Cytoplasm<br>Cytosol	Catalytic activity<br>Nucleotide binding
57	XP_003984106.1	Ras-Related Protein Rab-1A	22,7	8,957	5	23	Cell organization and biogenesis<br>Cellular component movement<br>Defense response<br>Metabolic process<br>Regulation of biological process<br>Response to stimulus<br>Transport	Golgi<br>Membrane	Catalytic activity<br>Nucleotide binding<br>Protein binding
58	AAA19458.1	MHC Class I Antigen	41	8,908	5	15	Response to stimulus	Membrane	Protein binding
59	XP_003992206.1	Ras-Related Protein Rab-5A	23,6	8,905	4	20	Regulation of biological process<br>Response to stimulus	Cytoplasm<br>Cytosol	Catalytic activity<br>Nucleotide binding
60	XP_003995861.1	Ras-Related Protein Rab-14	23,9	8,634	3	16	Cell organization and biogenesis<br>Defense response<br>Regulation of biological process<br>Response to stimulus<br>Transport	Cytosol<br>Endosome<br>Membrane	Catalytic activity<br>Nucleotide binding<br>Protein binding
61	AAA19457.1	MHC Class I Antigen	41	8,631	5	14	Response to stimulus	Membrane	Protein binding
62	XP_003986000.1	Complement Factor B	86,1	8,607	3	5	Metabolic process	Extracellular	Catalytic activity
63	XP_006939263.1	Cytoplasmic Aconitate Hydratase	98,4	8,506	5	5	Cellular homeostasis<br>Metabolic process<br>Regulation of biological process<br>Response to stimulus<br>Transport	Cytoplasm<br>Cytosol<br>Endoplasmic Reticulum<br>Golgi<br>Mitochondrion	Catalytic activity<br>Metal ion binding<br>RNA binding
64	XP_019667990.1	Catenin Delta-1 Isoform X1	107,4	8,431	5	6	Regulation of biological process	Cytoskeleton<br>Cytosol<br>Golgi<br>Membrane	Protein binding
65	XP_003993583.1	4F2 Cell-Surface Antigen Heavy Chain	58	8,341	4	8	Metabolic process	Cell Surface	Catalytic activity

66	XP_019678268.1	Ras-Related Protein Rab-2A Isoform X1	24,4	7,904	3	16	Response to stimulus<br>Transport	Cytosol<br>Endosome<br>Membrane	Catalytic activity<br>Nucleotide binding<br>Protein binding
67	XP_003998661.1	T-Complex Protein 1 Subunit Zeta Isoform X1	58	7,775	3	6	Metabolic process	Membrane	Catalytic activity<br>Nucleotide binding
68	XP_003987480.1	Ras-Related Protein Rab-2B Isoform X1	24,1	7,667	3	17	Regulation of biological process<br>Response to stimulus	Membrane	Catalytic activity<br>Nucleotide binding
69	XP_006932061.1	Band 4.1-Like Protein 2 Isoform X1	117,1	7,62	4	6	Cell organization and biogenesis	Cytoskeleton	Protein binding<br>Structural molecule activity
70	XP_019687754.1	60S Ribosomal Protein L4	47,3	7,498	3	7	Metabolic process	Ribosome	Structural molecule activity
71	XP_003988264.1	Neuropilin-1 Isoform X2	103,2	7,487	3	5	Cell differentiation<br>Cell growth<br>Cell organization and biogenesis<br>Cellular component movement<br>Development<br>Regulation of biological process<br>Response to stimulus<br>Transport	Membrane	Receptor activity
72	XP_003988551.1	GTPase Kras	21,4	7,457	3	19	Cell communication<br>Cell differentiation<br>Cell organization and biogenesis<br>Regulation of biological process<br>Response to stimulus	Cytoplasm<br>Membrane	Catalytic activity<br>Nucleotide binding<br>Protein binding
73	XP_003995204.1	ATP Synthase Subunit Alpha, Mitochondrial	59,7	7,36	4	7	Metabolic process<br>Regulation of biological process<br>Transport	Membrane<br>Mitochondrion	Catalytic activity<br>Nucleotide binding<br>Protein binding<br>RNA binding<br>Transporter activity
74	XP_011278541.1	Ras-Related Protein Rab-7A	23,5	7,231	4	20	Regulation of biological process<br>Response to stimulus	Membrane	Catalytic activity<br>Nucleotide binding
75	XP_004000896.2	Lysosome-Associated Membrane Glycoprotein 2 Isoform X1	51,5	7,065	4	6	Regulation of biological process<br>Cellular homeostasis	Membrane	Protein binding
76	XP_011277743.1	ADP/ATP Translocase 2	32,9	7,054	4	12	Regulation of biological process,<br>Respose to stimulus	Cytoplasm	Catalytic activity
77	XP_003992261.2	Catenin Beta-1 Isoform X1	85,5	7,013	4	6	Cell organization and biogenesis	Cytoskeleton<br>Cytosol<br>Golgi<br>Membrane	Protein binding<br>Signal transducer activity
78	XP_003982686.1	Ras-Related Protein Ral-A	23,5	6,997	3	19	Cell organization and biogenesis<br>Regulation of biological process<br>Response to stimulus	Cell Surface<br>Membrane	Catalytic activity<br>Nucleotide binding<br>Protein binding
79	XP_003984974.1	Fibrinogen Gamma Chain	49,4	6,962	3	7	Cell organization and biogenesis<br>Metabolic process<br>Regulation of biological process<br>Response to stimulus<br>Transport	Cell Surface	Protein binding<br>Structural molecule activity

80	XP_011286082.1	Transmembrane Protein 119	28,8	6,945	3	12	Cell differentiation<br>Regulation of biological process	Membrane	Receptor activity
81	XP_003993713.1	Ras-Related Protein Rab-1B	22,2	6,676	4	21	Cell organization and biogenesis<br>Regulation of biological process<br>Response to stimulus<br>Transport	Golgi<br>Membrane<br>Mitochondrion	Catalytic activity<br>Nucleotide binding
82	XP_003996965.3	Synaptic Vesicle Membrane Protein Vat-1 Homolog	42,5	6,577	4	11	Metabolic process	Membrane	Metal ion binding
83	XP_019677997.1	Dual Specificity Protein Kinase Clk2 Isoform X1	88,5	6,517	3	6	Metabolic process<br>Transport	Membrane	Catalytic activity<br>Nucleotide binding
84	XP_011287741.2	Galactokinase	36,3	6,371	4	10	Metabolic process	Cytoplasm	Catalytic activity
85	XP_006929262.1	Asparagine Synthetase	64,4	6,267	3	6	Cell communication<br>Metabolic process<br>Regulation of biological process<br>Response to stimulus	Cytosol	Catalytic activity<br>Nucleotide binding<br>Protein binding
86	XP_003991044.1	60 Kda Heat Shock Protein, Mitochondrial	60,9	6,196	3	5	Cell organization and biogenesis<br>Metabolic process<br>Regulation of biological process<br>Response to stimulus<br>Transport	Cytoplasm<br>Cytosol<br>Mitochondrion	Nucleotide binding<br>Protein binding
87	XP_003980861.1	Stress-70 Protein, Mitochondrial	73,6	6,179	3	5	Cell differentiation<br>Cell organization and biogenesis<br>Metabolic process<br>Regulation of biological process	Mitochondrion	Nucleotide binding<br>Protein binding<br>RNA binding
88	XP_011281217.1	Tropomyosin Alpha-1 Chain Isoform X3	28,6	5,882	3	8	Cell organization and biogenesis<br>Regulation of biological process<br>Response to stimulus	Cytoplasm<br>Cytosol	Protein binding
89	XP_019667650.1	Ras-Related Protein R-Ras2 Isoform X1	21,7	5,557	4	22	Cell differentiation<br>Regulation of biological process	Golgi<br>Membrane<br>Mitochondrion	Catalytic activity<br>Nucleotide binding
90	XP_006938678.1	14-3-3 Protein Eta	33,8	5,481	3	7	Metabolic process<br>Regulation of biological process	Cytoplasm	Protein binding<br>Receptor activity
91	XP_003984471.1	Ras-Related Protein Rab-10	22,5	5,454	3	17	Cell organization and biogenesis<br>Regulation of biological process<br>Response to stimulus<br>Transport	Endosome<br>Golgi<br>Membrane	Catalytic activity<br>Nucleotide binding<br>Protein binding
92	P61246.1	40S Ribosomal Protein S3A	29,6	5,363	3	10	Cell differentiation<br>Metabolic process	Cytoplasm<br>Cytosol<br>Nucleus<br>Ribosome	Structural molecule activity
93	XP_011285393.1	Tumor Necrosis Factor Receptor Superfamily Member 1A-Like	46,9	5,246	3	9	Regulation of biological process<br>Response to stimulus	Cell Surface	Protein binding
94	XP_011283649.1	Prolyl Endopeptidase Fap Isoform X1	87,7	5,231	3	6	Metabolic process	Cytoplasm<br>Cytosol	Protein binding
95	XP_019687854.1	Ras-Related Protein Rab-8B Isoform X1	30,8	4,203	3	10	Cell organization and biogenesis<br>Regulation of biological process<br>Response to stimulus<br>Transport	Cell Surface<br>Membrane	Protein binding

PROTEINS IN COMMON IN BOTH SAMPLES									
**Number**	**Accession**	**Description**	**MW [kDa]**	**Score**	**Peptides**	**Coverage [%]**	**Biological Process**	**Cellular Component**	**Molecular Function**
1	XP_006935598.1	Fibronectin isoform X4	262,2	790,156	80	45	Defense response<br>Regulation of biological process<br>Response to stimulus	Extracellular	Protein binding
2	XP_003991168.1	Fibronectin isoform X11	252,3	787,226	78	46	Defense response<br>Regulation of biological process<br>Response to stimulus	Extracellular	Protein binding
3	XP_006931911.1	Collagen alpha-1(XII) chain isoform X1	339	328,009	83	31	Cell differentiation<br>Cell organization and biogenesis<br>Metabolic process<br>Regulation of biological process<br>Response to stimulus<br>Transport	Endoplasmic Reticulum<br>Extracellular	Protein binding
4	XP_004001062.1	Filamin-A isoform X1	280,5	319,04	70	33	Cell organization and biogenesis<br>Coagulation<br>Development<br>Metabolic process<br>Regulation of biological process<br>Transport	Cytoskeleton	Protein binding
5	XP_003982813.1	Collagen alpha-2(I) chain	129,5	275,225	44	49	Cell organization and biogenesis<br>Regulation of biological process<br>Response to stimulus	Endoplasmic Reticulum<br>Extracellular	Protein binding<br>Structural molecule activity
6	XP_019694498.1	Collagen alpha-3(VI) chain	344,1	237,812	68	26	Cell organization and biogenesis<br>Regulation of biological process<br>Response to stimulus	Cytoplasm<br>Extracellular	Protein binding<br>Structural molecule activity
7	XP_006940721.2	Actin, cytoplasmic 2	41,8	159,38	18	55	Cell organization and biogenesis	Cytoskeleton<br>Membrane<br>Nucleus	Nucleotide binding<br>Protein binding<br>Structural molecule activity
8	XP_003988180.1	Vimentin	53,6	154,844	30	50	Cell differentiation<br>Cell organization and biogenesis<br>Metabolic process<br>Regulation of biological process<br>Response to stimulus	Cytoplasm<br>Cytoskeleton<br>Cytosol<br>Membrane	Protein binding<br>RNA binding<br>Structural molecule activity
9	XP_019671966.1	Tenascin isoform X1	250,7	131,565	31	21	Cell communication<br>Cell differentiation<br>Cell organization and biogenesis<br>Metabolic process	Extracellular	Protein binding

							Regulation of biological process<br>Response to stimulus		
10	XP_019678170.1	Plectin	480,6	129,339	55	15	Cell organization and biogenesis<br>Regulation of biological process	Cytoplasm<br>Cytoskeleton<br>Cytosol<br>Membrane	Protein binding
11	XP_019668863.1	Vinculin isoform X1	123,9	118,695	30	32	Cell organization and biogenesis<br>Regulation of biological process	Cytoskeleton<br>Membrane	Protein binding<br>Structural molecule activity
12	XP_019678982.1	Filamin-B isoform X1	280,8	114,846	42	21	Cell differentiation<br>Cell organization and biogenesis	Cytoskeleton	Protein binding
13	XP_011281379.1	Thrombospondin-1	129,5	106,823	26	21	Cell organization and biogenesis<br>Cellular component movement<br>Defense response<br>Metabolic process<br>Regulation of biological process<br>Response to stimulus	Extracellular	Metal ion binding<br>Protein binding
14	XP_006936975.1	Serpin H1	46,4	103,768	18	39	Cell organization and biogenesis<br>Metabolic process<br>Regulation of biological process	Cytoplasm<br>Endoplasmic<br>Reticulum<br>Membrane	Enzyme regulator activity<br>Protein binding<br>RNA binding
15	XP_003989518.1	Alpha-enolase isoform X1	47,3	100,635	19	49	Metabolic process<br>Regulation of biological process<br>Response to stimulus	Cell Surface<br>Cytoplasm<br>Cytosol<br>Membrane<br>Nucleus	Catalytic activity<br>DNA binding<br>Metal ion binding<br>Protein binding<br>RNA binding
16	XP_019671727.1	Talin-1 isoform X1	271,3	98,759	28	15	Cell organization and biogenesis	Cytoskeleton	protein binding<br>structural molecule activity
17	XP_006929424.1	Filamin-C isoform X1	292,5	90,929	27	13	Cell organization and biogenesis<br>Development	Cytoskeleton	Protein binding
18	XP_003991681.1	Coiled-coil domain-containing protein 80	108	88,924	19	24	Cell organization and biogenesis<br>Regulation of biological process	Cytoskeleton<br>Cytosol<br>Endoplasmic<br>Reticulum<br>Membrane<br>Golgi	Protein binding
19	XP_019691058.1	Myosin-9	220,7	81,798	35	17	Cell differentiation<br>Cell organization and biogenesis<br>Cellular component movement<br>Metabolic process<br>Regulation of biological process<br>Transport	Cytoplasm<br>Cytosol<br>Extracellular	Protein binding
20	XP_003992527.1	Heat Shock Cognate 71 Kda Protein	70,9	75,311	20	40	Cell organization and biogenesis	Membrane	Nucleotide binding
21	XP_003992442.1	Transgelin	22,6	67,95	13	60	Cell differentiation<br>Development	Membrane	Protein binding

22	XP_004000714.1	Phosphoglycerate kinase 1	44,6	67,717	13	37	Metabolic process	Cytosol	Catalytic activity<br>Nucleotide binding
23	XP_004000620.1	Moesin	67,8	66,125	25	35	Cell differentiation<br>Cell organization and biogenesis<br>Cell proliferation<br>Cellular component movement<br>Regulation of biological process<br>Response to stimulus	Cell Surface<br>Cytoplasm<br>Cytoskeleton<br>Cytosol<br>Membrane<br>Nucleus	Protein binding<br>RNA binding
24	XP_003998733.1	Fructose-bisphosphate aldolase A	39,5	64,311	12	35	Metabolic process	Cytosol	Catalytic activity
25	XP_011289490.1	Lamin	73,9	63,07	23	38	Cell organization and biogenesis<br>Regulation of biological process	Cytoskeleton<br>Cytosol<br>Nucleus	Structural molecule activity
26	XP_019690668.1	Prolow-density lipoprotein receptor-related protein 1 isoform X1	506,1	62,749	20	5	Metabolic process<br>Response to stimulus	Membrane	Receptor activity
27	XP_019685525.1	WD repeat-containing protein 1, partial	69,4	62,393	15	37	Cell differentiation<br>Cell organization and biogenesis<br>Cellular component movement<br>Metabolic process<br>Regulation of biological process	Cytoskeleton	Protein binding
28	XP_003981179.1	Versican Core Protein Isoform X1	371,7	60,238	16	5	Cell differentiation	Extracellular<br>Membrane	Metal ion binding<br>Protein binding<br>Structural molecule activity
29	XP_019667711.1	L-lactate dehydrogenase A chain isoform X1	39,7	59,957	15	37	Metabolic process	Cytoplasm	Catalytic activity
30	XP_011283275.1	Periostin isoform X3	89,9	53,85	14	21	Cell communication<br>Cell organization and biogenesis<br>Regulation of biological process<br>Response to stimulus	Extracellular<br>Golgi	Protein binding
31	AMJ27376.1	LTBP2	195,7	51,945	11	9	Response to stimulus	Cell Surface<br>Membrane	Metal ion binding
32	XP_003985935.1	Tubulin beta chain	49,6	50,217	13	38	Cell organization and biogenesis	Cytoplasm<br>Cytoskeleton<br>Nucleus	Catalytic activity<br>Nucleotide binding<br>Protein binding<br>Structural molecule activity
33	XP_011289183.1	Peroxiredoxin-6	25	49,251	12	42	Metabolic process	Cytosol<br>Mitochondrion<br>Nucleus	Antioxidant activity<br>Catalytic activity
34	XP_011280387.2	Histone H3.1 isoform X2	24,3	46,54	13	36	Cell organization and biogenesis<br>Regulation of biological process	Nucleus	DNA binding
35	XP_003981745.1	Elongation factor 2	95,3	44,856	13	16	Metabolic process	Nucleus	Catalytic activity<br>Nucleotide binding

36	XP_003998575.2	Plasminogen activator inhibitor 1	45,2	44,639	13	39	Defense response<br>Regulation of biological process<br>Response to stimulus	Extracellular	Enzyme regulator activity<br>Protein binding
37	XP_006939520.1	78 kDa glucose-regulated protein	72,2	44,414	16	28	Cell organization and biogenesis	Endoplasmic reticulum	Catalytic activity
38	XP_011287043.1	Tubulin Beta-4B Chain	49,8	44,077	12	28	Cell organization and biogenesis	Cytoplasm<br>Cytoskeleton<br>Nucleus	Catalytic activity<br>Nucleotide binding<br>Structural molecule activity
39	XP_011286873.1	Transitional endoplasmic reticulum ATPase isoform X1	89,2	43,749	12	18	Metabolic process<br>Response to stimulus	Endoplasmic reticulum	Catalytic activity<br>Nucleotide binding<br>RNA binding
40	XP_011289352.2	Laminin subunit gamma-1	172,4	43,096	12	11	Cell differentiation<br>Cell organization and biogenesis<br>Cellular component movement<br>Metabolic process<br>Regulation of biological process<br>Transport	Extracellular	Catalytic activity<br>Motor activity<br>Nucleotide binding<br>Protein binding<br>Structural molecule activity<br>Transporter activity
41	XP_003987117.1	Annexin A2	38,6	41,97	10	30	Cell organization and biogenesis<br>Metabolic process<br>Regulation of biological process<br>Coagulation<br>Transport	Extracellular	Metal ion binding
42	XP_003988041.1	Heat Shock Protein Hsp 90-Alpha	84,7	41,891	14	19	Metabolic process<br>Response to stimulus	Membrane	Nucleotide binding<br>Protein binding
43	XP_019689513.1	Dihydropyrimidinase-related protein 3	71,1	38,92	12	27	Cell organization and biogenesis<br>Cellular component movement<br>Regulation of biological process	Cytosol	Catalytic activity
44	XP_003988693.2	Tubulin alpha-1A chain	50,1	37,804	10	28	Cell organization and biogenesis	Cytoplasm<br>Cytoskeleton<br>Nucleus	Catalytic activity<br>Nucleotide binding<br>Structural molecule activity
45	XP_019673407.1	Keratin, type I cytoskeletal 10 isoform X1	87,8	37,42	13	16	Cell organization and biogenesis<br>Cellular component movement<br>Metabolic process	Cytoskeleton<br>Membrane<br>Nucleus	Structural molecule activity<br>Transporter activity
46	XP_019685880.1	Tubulin Beta Chain-Like Isoform X2	55,3	36,85	9	19	Cell organization and biogenesis	Cytoplasm<br>Cytoskeleton<br>Nucleus	Protein binding<br>Structural molecule activity
47	XP_003986248.1	Heat Shock Protein Hsp 90-Beta	83,2	36,821	12	17	Cell organization and biogenesis<br>Metabolic process<br>Regulation of biological process<br>Response to stimulus	Cytoplasm<br>Cytosol<br>Extracellular<br>Membrane<br>Mitochondrion<br>Nucleus	Enzyme regulator activity<br>Nucleotide binding<br>Protein binding<br>RNA binding
48	XP_019669655.1	Serine protease HTRA1	41,8	34,929	7	22	Metabolic process	Extracellular	Catalytic activity
49	XP_003988789.1	Keratin, type II cytoskeletal 1	64,7	34,627	7	10	Defense response<br>Metabolic process<br>Regulation of biological process<br>Response to stimulus	Cytoskeleton<br>Membrane<br>Nucleus	Structural molecule activity

50	NP_001009326.1	Elongation factor 1-alpha 1	50,1	34,444	9	24	Metabolic process<br>Response to stimulus	Cytoplasm<br>Cytoskeleton<br>Cytosol<br>Membrane<br>Nucleus	Catalytic activity<br>Nucleotide binding<br>Protein binding<br>RNA binding
51	XP_011284222.1	Peptidyl-prolyl cis-trans isomerase A	17,9	34,221	8	49	Metabolic process	Extracellular	Catalytic activity
52	XP_006930945.1	Annexin A5	35,9	34,126	11	27	Cell organization and biogenesis<br>Metabolic process<br>Regulation of biological process<br>Coagulation<br>Transport	Extracellular	Metal ion binding
53	NP_001009961.1	Serum albumin precursor	68,6	32,367	5	7	Cell communication<br>Regulation of biological process<br>Response to stimulus<br>Transport	Extracellular	DNA binding<br>Metal ion binding
54	XP_011281069.1	Ras GTPase-activating-like protein IQGAP1	185,5	31,69	13	10	Regulation of biological process	Cytoskeleton	Protein binding
55	XP_011279577.1	EMILIN-1	107,5	30,721	12	15	Regulation of biological process	Extracellular	Protein binding
56	XP_004000473.1	Ubiquitin-like modifier-activating enzyme 1	117,8	30,682	11	14	Metabolic process<br>Response to stimulus	Cytoplasm<br>Cytosol<br>Membrane<br>Mitochondrion<br>Nucleus	Catalytic activity<br>Nucleotide binding<br>RNA binding
57	XP_019673150.1	Clathrin Heavy Chain 1 Isoform X1	192,4	29,744	13	9	Cell organization and biogenesis<br>transport	Cytoplasm	Protein binding
58	XP_003990084.1	Peroxiredoxin-1	22,1	27,976	11	47	Cell proliferation<br>Cellular homeostasis<br>Defense response<br>Metabolic process<br>Regulation of biological process<br>Response to stimulus	Cytoplasm<br>Mitochondrion<br>Nucleus	Antioxidant activity<br>Catalytic activity<br>Protein binding<br>RNA binding
59	XP_003994311.3	Phosphoglycerate mutase 1	28,8	27,485	7	35	Metabolic process<br>Regulation of biological process	Cytoplasm<br>Cytosol<br>Membrane	Catalytic activity<br>Protein binding
60	XP_003984253.1	Macrophage-capping protein	38,9	26,664	8	33	Cell organization and biogenesis<br>Regulation of biological process	Nucleus	Protein binding
61	XP_003989530.1	6-phosphogluconate dehydrogenase, decarboxylating	53,2	26,648	7	20	Metabolic process	Nucleus	Catalytic activity
62	XP_003995862.1	Gelsolin	85,8	25,742	9	13	Cell death<br>Cell organization and biogenesis<br>Metabolic process<br>Regulation of biological process<br>Response to stimulus<br>Transport	Cytoskeleton	Structural molecule activity

63	XP_003986732.1	Ezrin	69,3	25,611	9	13	Cell organization and biogenesis<br>Metabolic process<br>Regulation of biological process<br>Response to stimulus<br>Transport	Cytoplasm	Protein binding
64	XP_003999021.1	Hemoglobin subunit alpha-like	15,4	24,99	3	18	Transport	Extracellular	Metal ion binding<br>Transporter activity
65	XP_011283955.1	Glia-derived nexin	44	24,72	8	25	Cell organization and biogenesis<br>Regulation of biological process<br>Response to stimulus<br>Transport	Cytosol	Receptor activity
66	XP_003982988.2	Glycine--tRNA ligase, partial	81	24,505	8	13	Metabolic process	Cytosol	Catalytic activity<br>Nucleotide binding
67	XP_011282630.2	Fibulin-1	78	23,297	7	9	Cell organization and biogenesis<br>Metabolic process<br>Regulation of biological process<br>Response to stimulus	Extracellular	Metal ion binding<br>Receptor activity
68	XP_006933272.1	Rab GDP dissociation inhibitor beta	48	22,868	6	14	Metabolic process<br>Regulation of biological process<br>Response to stimulus<br>Transport	Cytoplasm	Catalytic activity<br>Enzyme regulator activity<br>RNA binding
69	XP_006937472.1	Neuroblast Differentiation-Associated Protein AHNAK	626,8	22,407	11	11	Cell organization and biogenesis<br>Regulation of biological process	Nucleus	RNA binding<br>Structural molecule activity
70	XP_011283013.1	Adenylyl cyclase-associated protein 1 isoform X2	51,4	22,21	6	17	Cell organization and biogenesis<br>Regulation of biological process<br>Response to stimulus	Cytoskeleton	Protein binding
71	XP_003989158.1	Lumican	38,4	22,177	7	17	Cell organization and biogenesis<br>Regulation of biological process	Extracellular	Protein binding
72	XP_003980811.1	Heat shock 70 kDa protein 4 isoform X1	94,3	22,168	8	12	Cell organization and biogenesis<br>Transport	Cytosol	Nucleotide binding
73	XP_003996471.1	14-3-3 protein epsilon isoform X1	29,2	21,439	7	29	Metabolic process<br>Regulation of biological process	Cytoplasm	Nucleotide binding
74	XP_003988788.1	Keratin, type II cytoskeletal 2 epidermal	67,4	21,39	7	12	Cell organization and biogenesis<br>Cell proliferation<br>Cellular component movement<br>Metabolic process	Cytoskeleton<br>Membrane<br>Nucleus	Protein binding<br>Structural molecule activity
75	XP_003999257.1	Antithrombin-III	52,4	21,279	6	13	Regulation of biological process	Extracellular	Enzyme regulator activity<br>Protein binding
76	XP_004001337.3	Keratin, Type II Cytoskeletal 5 Isoform X1	62,7	20,907	9	11	Metabolic process	Cytoskeleton<br>Membrane<br>Nucleus	Structural molecule activity
77	XP_003988134.1	Inter-alpha-trypsin inhibitor heavy chain H2	106,3	19,978	6	8	Metabolic process	Extracellular	Enzyme regulator activity
78	XP_003993558.1	Elongation factor 1-gamma	50	19,805	8	19	Metabolic process	Nucleus<br>Endoplasmic Reticulum	Protein binding<br>RNA binding

79	XP_003995053.1	Tubulin Beta-6 Chain Isoform X1	49,9	19,404	6	10	Cell organization and biogenesis	Cytoplasm<br>Cytoskeleton<br>Nucleus	Catalytic activity<br>Nucleotide binding<br>Structural molecule activity
80	XP_006942940.1	Fibromodulin	43,1	19,356	3	11	Cell organization and biogenesis	Extracellular	Protein binding
81	XP_006929172.2	Laminin subunit beta-1	197,2	18,583	8	5	Cell differentiation<br>Cell organization and biogenesis<br>Cellular component movement<br>Metabolic process<br>Regulation of biological process<br>Transport	Extracellular	Catalytic activity<br>Motor activity<br>Nucleotide binding<br>Protein binding<br>Structural molecule activity<br>Transporter activity
82	XP_003991166.1	Bifunctional purine biosynthesis protein PURH	64,8	18,484	6	13	Metabolic process	Cytosol<br>Extracellular<br>Membrane	Catalytic activity
83	XP_003996224.1	Eukaryotic Initiation Factor 4A-I	46,1	18,139	8	23	Metabolic process<br>Regulation of biological process	Cytoplasm<br>Membrane	Catalytic activity<br>DNA binding<br>Nucleotide binding<br>RNA binding
84	XP_003988536.1	L-lactate dehydrogenase B chain	36,6	17,714	8	25	Metabolic process	Cytoplasm	Catalytic activity
85	XP_003996286.2	Profilin-1	11	17,7	4	52	Cell organization and biogenesis<br>Regulation of biological process	Extracellular	Protein binding
86	XP_003986736.1	T-complex protein 1 subunit alpha	60,2	17,634	4	9	Metabolic process<br>Regulation of biological process<br>Transport	Cytoskeleton<br>Cytosol	Nucleotide binding
87	XP_003983647.1	Adenosylhomocysteinase Isoform X1	47,7	17,174	6	16	Metabolic process<br>Transport	Cytosol	Catalytic activity<br>Nucleotide binding
88	XP_003988038.1	Tryptophan--tRNA ligase, cytoplasmic	53,4	17,089	7	23	Metabolic process<br>Regulation of biological process	Cytoplasm<br>Cytosol<br>Nucleus	Catalytic activity<br>Enzyme regulator activity<br>Nucleotide binding<br>Protein binding
89	XP_003985991.1	Heat Shock 70 Kda Protein 1-Like	70,3	16,88	6	12	Cell organization and biogenesis<br>Metabolic process	Cytosol	Nucleotide binding<br>Protein binding
90	XP_003998588.1	14-3-3 Protein Gamma	28,3	16,159	6	21	Metabolic process<br>Regulation of biological process	Cytoplasm	Protein binding
91	XP_006933527.1	Triosephosphate isomerase	30,8	15,364	5	21	Metabolic process	Cytosol	Catalytic activity
92	XP_003985985.1	Chloride intracellular channel protein 1	27	15,348	4	29	Metabolic process<br>Regulation of biological process<br>Transport	Cytoplasm<br>Membrane<br>Mitochondrion<br>Nucleus	Catalytic activity<br>Protein binding<br>Transporter activity
93	XP_019694553.1	Septin-2 isoform X3	43,8	15,091	4	18	Regulation of biological process<br>Response to stimulus	Cytoskeleton<br>Membrane	Nucleotide binding<br>Protein binding
94	XP_011280034.1	Septin-11 isoform X1	49,8	15,038	6	17	Regulation of biological process<br>Response to stimulus	Cytoskeleton<br>Membrane	Nucleotide binding

95	XP_003985479.1	UDP-Glucose 6-Dehydrogenase	55	14,996	7	17	Metabolic process	Cytosol<br>Nucleus	Catalytic activity<br>Nucleotide binding
96	XP_003992907.1	Hemoglobin Subunit Epsilon-2 Isoform X1	16,4	14,47	3	16	Transport<br>Metabolic procees	Extracellular	Metal ion binding<br>Transporter activity
97	XP_019682012.1	14-3-3 Protein Beta/Alpha Isoform X1	30	14,413	5	19	Metabolic process<br>Regulation of biological process	Cytoplasm	Protein binding
98	XP_003999651.1	Transgelin-2	22,4	14,036	5	34	Cell differentiation	Membrane	protein binding
99	XP_011279736.1	Dihydropyrimidinase-related protein 2 isoform X1	73,5	13,865	6	9	Cell organization and biogenesis<br>Cellular component movement<br>Regulation of biological process	Cytoskeleton<br>Cytosol	Catalytic activity
100	XP_019675162.1	Glucose-6-phosphate isomerase	55,3	13,84	4	10	Metabolic process	Membrane	Catalytic activity
101	XP_019672542.1	Protein disulfide-isomerase	58,8	13,504	5	11	Regulation of biological process	Membrane<br>Cytoskeleton<br>Endoplasmic Reticulum<br>Cytosol	Catalytic activity
102	XP_006930844.1	Palladin Isoform X1	129,2	13,369	5	5	Cell organization and biogenesis,<br>Cellular component movement	Cytoskeleton	Protein binding
103	XP_006936000.1	T-complex protein 1 subunit theta isoform X1	60,3	12,353	4	9	Metabolic process<br>Regulation of biological process<br>Transport	Cytoskeleton	Nucleotide binding
104	XP_006931645.1	Complement C4-A	191,8	12,327	6	4	Regulation of biological process<br>Response to stimulus	Extracellular	Enzyme regulator activity
105	XP_011278191.1	Polyubiquitin-B-Like, Partial	21,2	12,19	3	42	Metabolic process	Nucleus	Protein binding
106	XP_019673441.1	Keratin, Type I Cytoskeletal 14 Isoform X2	60,6	11,64	7	12	Defense response<br>Metabolic process<br>Regulation of biological process<br>Response to stimulus	Cytoskeleton<br>Membrane<br>Nucleus	Structural molecule activity<br>Transporter activity
107	XP_011278568.1	Uridine phosphorylase 1 isoform X1	34,6	10,96	3	18	Metabolic process	Cytosol	Catalytic activity
108	XP_003995475.1	Phosphoserine aminotransferase	40,4	10,316	4	12	Metabolic process	Cytoplasm<br>Cytosol	Catalytic activity
109	XP_003984089.1	UTP--Glucose-1-Phosphate Uridylyltransferase Isoform X1	56,9	10,303	5	10	Metabolic process	Cytosol	Catalytic activity
110	XP_019691975.1	Chloride intracellular channel protein 4	26,8	10,173	3	19	Regulation of biological process<br>Response to stimulus	Nucleus<br>Cytoskeleton<br>Membrane	Metal ion binding
111	XP_019690982.1	Endoplasmin	92,4	9,686	4	5	Cell organization and biogenesis<br>Metabolic process<br>Regulation of biological process<br>Response to stimulus<br>Transport	Cytosol<br>Endoplasmic Reticulum<br>Membrane	Nucleotide binding<br>Protein binding<br>RNA binding

								Nucleus<br>Organelle Lumen	
112	XP_003998507.1	Actin-related protein 2/3 complex subunit 1B	41	9,466	4	13	Cell organization and biogenesis<br>Regulation of biological process	Cytoskeleton	Protein binding<br>Structural molecule activity
113	XP_003987704.1	Galectin-3	28,8	9,385	5	22	Cell differentiation<br>Cellular component movement<br>Regulation of biological process<br>Response to stimulus	Cytoplasm<br>Extracellular<br>Membrane<br>Nucleus	Protein binding<br>RNA binding
114	XP_019684790.1	PDZ and LIM domain protein 5 isoform X15	67,4	9,284	4	9	Development<br>Cell organization and biogenesis	Cytoskeleton	Protein binding
115	XP_003988864.1	Keratin, Type II Cytoskeletal 2 Oral	64,9	9,186	4	4	Cell differentiation<br>Metabolic process	Cytoskeleton<br>Membrane<br>Nucleus	Catalytic activity<br>Motor activity<br>Protein binding<br>Structural molecule activity
116	XP_006934756.1	Alcohol dehydrogenase [NADP(+)]	36,7	8,944	4	14	Metabolic process	Cytosol<br>Membrane	catalytic activity
117	NP_001265776.1	Peptidyl-prolyl cis-trans isomerase B	23,8	8,878	4	17	Metabolic process<br>Regulation of biological process	Endoplasmic Reticulum<br>Membrane<br>Nucleus	Catalytic activity<br>Protein binding<br>RNA binding
118	XP_003985337.1	Alpha-fetoprotein	68,7	8,753	5	7	Metabolic process<br>Regulation of biological process<br>Reproduction<br>Response to stimulus<br>Transport	Cytoplasm<br>Cytosol<br>Extracellular	Metal ion binding
119	XP_011285993.1	GTP-binding nuclear protein Ran	26,4	8,288	4	19	Regulation of biological process<br>Response to stimulus	Cytosol<br>Nucleus	Nucleotide binding
120	XP_006940265.1	Nucleoside diphosphate kinase A	17,1	8,244	4	35	Metabolic process	Cytosol<br>Membrane<br>Nucleus	Nucleotide binding<br>Transcription regulator activity
121	XP_003999670.1	T-complex protein 1 subunit gamma isoform X1	60,5	8,209	3	6	Cell organization and biogenesis<br>Metabolic process<br>Regulation of biological process<br>Transport	Cytoskeleton<br>Cytosol	Nucleotide binding
122	XP_019690632.1	Myosin Light Polypeptide 6 Isoform X1	26	8,101	3	13	Cell organization and biogenesis<br>Cellular component movement<br>Regulation of biological process	Cytoskeleton<br>Cytosol<br>Extracellular	Metal ion binding<br>Structural molecular activity
123	XP_011278740.1	Heterogeneous nuclear ribonucleoproteins A2/B1	37,4	7,865	4	17	Metabolic process<br>Regulation of biological process<br>Transport	Cytoplasm<br>Membrane<br>Nucleus<br>Spliceosomal Complex	DNA binding<br>RNA binding
124	XP_006928916.1	Inter-alpha-trypsin inhibitor heavy chain H3	99,3	7,809	3	4	Metabolic process	Extracellular	Enzyme regulator activity
125	XP_019673333.1	Importin subunit beta-1	97,2	7,485	5	6	Transport	Nucleus	Protein binding

126	XP_003989084.1	T-complex protein 1 subunit beta	57,4	6,763	3	9	Cell organization and biogenesis<br>Metabolic process<br>Regulation of biological process<br>Transport	Cytoskeleton<br>Cytosol	Nucleotide binding
127	XP_003984741.1	Lysyl oxidase homolog 2	86,8	6,611	4	5	Cell organization and biogenesis<br>Cell proliferation<br>Cellular component movement<br>Metabolic process<br>Regulation of biological process<br>Response to stimulus<br>Transport	Membrane	Catalytic activity<br>Metal ion binding<br>Receptor activity
128	XP_003995546.2	40S ribosomal protein S6	35,5	6,556	3	11	Metabolic process	Ribosome	Structural molecule activity
129	XP_003989294.1	Galectin-1	14,7	6,08	3	24	Cell differentiation<br>Regulation of biological process<br>Response to stimulus	Extracellular	RNA binding<br>Signal transducer activity
130	XP_003990566.2	d-3-phosphoglycerate dehydrogenase	56,5	5,666	3	8	Metabolic process	Cytoplasm	Catalytic activity<br>Nucleotide binding
131	XP_003993693.1	Cofilin-1	18,5	5,468	3	16	Cell organization and biogenesis	Cytoskeleton	Protein binding
132	XP_003993600.1	Fermitin Family Homolog 3	75,3	5,051	3	5	Cell organization and biogenesis<br>Regulation of biological process<br>Response to stimulus	Cytosol<br>Endosome<br>Membrane	Catalytic activity<br>Nucleotide binding<br>Protein binding
133	AAB01670.1	Ribosomal protein S4, partial	29,4	4,71	3	10	Metabolic process	Ribosome	Structural molecule activity
