# Supplementary material for: Proteomic Analysis of the Secretome and Exosomes of Feline Adipose-Derived Mesenchymal Stem Cells
Source: Animals (Basel). 2021 Jan 24;11(2):295. doi: 10.3390/ani11020295 (PMC7912403; doi:10.3390/ani11020295)
Supplement: Supplementary file 1 [file animals-11-00295-s001.zip › S2 Table_Abbreviations for secretome and exosome proteins.pdf]

S2 Table. List of abbreviation proteins.

A. fAd-MSC SECRETOME	
**Abbreviation**	**Name**
ACTA2	Actin alpha 2; Belongs to the actin family (377 aa)
ACTB	Actin gamma 1; Belongs to the actin family (375 aa)
ACTN1	Actinin alpha 1 (872 aa)
ACTR1A	ARP1 actin-related protein 1 homolog A; Belongs to the actin family (376 aa)
AFP	Alpha-fetoprotein (609 aa)
AKR1A1	Aldo-keto reductase family 1, member A1 (aldehyde reductase) (325 aa)
ALB	Serum albumin (607 aa)
ALDOA	Aldolase A, fructose-bisphosphate (701 aa)
ALDOC	Aldolase C, fructose-bisphosphate (364 aa)
ARHGDIA	Uncharacterized protein (204 aa)
ARPC1B	Actin-related protein 2/3 complex subunit; Functions as component of the Arp2/3 complex which is involved in regulation of actin polymerization and together with an activating nucleation-promoting factor (NPF) mediates the formation of branched actin networks (372 aa)
ATIC	Uncharacterized protein (592 aa)
BGN	Biglycan; May be involved in collagen fiber assembly (372 aa)
CALU	Calumenin (241 aa)
CAPG	Uncharacterized protein (349 aa)
CAPZA1	Capping actin protein of muscle Z-line alpha subunit 1; F-actin-capping proteins bind in a Ca(2+)-independent manner to the fast growing ends of actin filaments (barbed end) thereby blocking the exchange of subunits at these ends. Unlike other capping proteins (such as gelsolin and severin), these proteins do not sever actin filaments (288 aa)
CCDC80	Coiled-coil domain containing 80 (956 aa)

CCT2	Chaperonin containing TCP1 subunit 2; Molecular chaperone; assists the folding of proteins upon ATP hydrolysis (535 aa)
CCT3	T-complex protein 1 subunit gamma; Molecular chaperone; assists the folding of proteins upon ATP hydrolysis (577 aa)
CCT4	T-complex protein 1 subunit delta; Molecular chaperone; assists the folding of proteins upon ATP hydrolysis (420 aa)
CFL1	Uncharacterized protein; Belongs to the actin-binding proteins ADF family (223 aa)
CLIC1	Chloride intracellular channel 1; Belongs to the chloride channel CLIC family (241 aa)
CNN2	Calponin; Thin filament-associated protein that is implicated in the regulation and modulation of smooth muscle contraction. It is capable of binding to actin, calmodulin, troponin C and tropomyosin. The interaction of calponin with actin inhibits the actomyosin Mg-ATPase activity (307 aa)
COL1A2	Collagen, type I, alpha 2 (1367 aa)
COL3A1	Collagen, type III, alpha 1 (1466 aa)
COL5A2	Collagen, type V, alpha 2 (1494 aa)
COMP	Cartilage oligomeric matrix protein (756 aa)
CORO1C	Coronin, actin binding protein, 1C; Belongs to the WD repeat coronin family (474 aa)
CTSB	Cathepsin B; Belongs to the peptidase C1 family (339 aa)
DCN	Decorin; May affect the rate of fibrils formation (360 aa)
EEF1A1	Elongation factor 1-alpha 1; Belongs to the TRAFAC class translation factor GTPase superfamily. Classic translation factor GTPase family. EF-Tu/EF-1A subfamily (462 aa)
EEF1G	Eukaryotic translation elongation factor 1 gamma (490 aa)
EEF2	Uncharacterized protein; Eukaryotic translation elongation factor 2 (858 aa)
EIF4A1	Eukaryotic translation initiation factor 4A1; Belongs to the DEAD box helicase family (406 aa)
EIF4A2	Eukaryotic translation initiation factor 4A2; Belongs to the DEAD box helicase family (407 aa)
ENO1	Enolase 1, (alpha) (434 aa)
ENSFCAG00000007301	Adenosylhomocysteinase (432 aa)
ENSFCAG00000007862	annotation not available (342 aa)
ENSFCAG00000012460	Adenylyl cyclase-associated protein ; Belongs to the CAP family (475 aa)

ENSFCAG00000012639	L-lactate dehydrogenase ; Belongs to the LDH/MDH superfamily (334 aa)
ENSFCAG00000015735	L-lactate dehydrogenase ; Belongs to the LDH/MDH superfamily (361 aa)
ENSFCAG00000025894	Uncharacterized protein; Belongs to the globin family (149 aa)
EZR	Ezrin (603 aa)
FLNA	Filamin A, alpha (2651 aa)
FMOD	Fibromodulin (376 aa)
FSTL1	Follistatin-like 1 (307 aa)
GANAB	Glucosidase, alpha; Belongs to the glycosyl hydrolase 31 family (966 aa)
GAPD	Glyceraldehyde-3-phosphate dehydrogenase (333 aa)
GDI1	Rab GDP dissociation inhibitor; Regulates the GDP/GTP exchange reaction of most RAB proteins by inhibiting the dissociation of GDP from them, and the subsequent binding of GTP (447 aa)
GDI2	Rab GDP dissociation inhibitor; Regulates the GDP/GTP exchange reaction of most RAB proteins by inhibiting the dissociation of GDP from them, and the subsequent binding of GTP (423 aa)
GOLM1	Golgi membrane protein 1 (406 aa)
GREM1	Gremlin 1, DAN family BMP antagonist (184 aa)
GSN	Gelsolin (751 aa)
HBA	Hemoglobin subunit alpha; Involved in oxygen transport from the lung to the various peripheral tissues (142 aa)
HEXB	Beta-hexosaminidase subunit beta; Belongs to the glycosyl hydrolase 20 family (458 aa)
HIST1H1D	Histone cluster 1, H1d (222 aa)
HIST1H2BB	Histone cluster 1, H2bb (126 aa)
HNRNPA1	Heterogeneous nuclear ribonucleoprotein A1 (373 aa)
HNRNPA2B1	Heterogeneous nuclear ribonucleoprotein A2/B1 (353 aa)
HSP90AA1	Heat shock protein 90kDa alpha (cytosolic), class A member 1 (745 aa)
HSP90AB1	Heat shock protein 90kDa alpha (cytosolic), class B member 1 (724 aa)
HSP90B1	Heat shock protein 90kDa beta (Grp94), member 1 (804 aa)

HSPA1L	Heat shock 70kDa protein 1-like (641 aa)
HSPA4	Heat shock 70kDa protein 4 (840 aa)
HSPA8	Heat shock 70kDa protein 8 (646 aa)
HSPB1	Heat shock 27kDa protein 1; Belongs to the small heat shock protein (HSP20) family (205 aa)
IDH1	Isocitrate dehydrogenase 1 (NADP+), soluble; Belongs to the isocitrate and isopropylmalate dehydrogenases family (414 aa)
ITIH2	Inter-alpha-trypsin inhibitor heavy chain 2 (950 aa)
KRT1	Keratin 1; Belongs to the intermediate filament family (630 aa)
KRT2	Keratin 2; Belongs to the intermediate filament family (666 aa)
KRT76	Keratin 76; Belongs to the intermediate filament family (617 aa)
LAMB1	Laminin, beta 1 (1786 aa)
LAP3	Leucine aminopeptidase 3 (520 aa)
LGALS1	Galectin; Lectin, galactoside-binding, soluble, 1 (135 aa)
LGALS3	Galectin; Lectin, galactoside-binding, soluble, 3 (278 aa)
LOXL2	Lysyl oxidase-like 2 (773 aa)
LPL	Lipoprotein lipase; Belongs to the AB hydrolase superfamily. Lipase family (478 aa)
LUM	Lumican (339 aa)
MDH1	Malate dehydrogenase, cytoplasmic (334 aa)
MMP19	Matrix metallopeptidase 19 (529 aa)
MMP2	Matrix metallopeptidase 2 (gelatinase A, 72kDa gelatinase, 72kDa type IV collagenase); Belongs to the peptidase M10A family (618 aa)
MSN	Moesin (577 aa)
NID1	Nidogen 1 (1244 aa)
PDIA3	Protein disulfide isomerase family A, member 3 (505 aa)
PDIA6	Protein disulfide isomerase family A, member 6; Belongs to the protein disulfide isomerase family (445 aa)

PGAM4	Phosphoglycerate mutase family member 4; Belongs to the phosphoglycerate mutase family. BPG- dependent PGAM subfamily (254 aa)
PGD	6-phosphogluconate dehydrogenase, decarboxylating; Belongs to the 6-phosphogluconate dehydrogenase family (483 aa)
PGK1	Phosphoglycerate kinase (417 aa)
PLOD1	Procollagen-lysine, 2-oxoglutarate 5-dioxygenase 1 (727 aa)
PLXDC2	Plexin domain containing 2 (529 aa)
PPIB	Peptidyl-prolyl cis-trans isomerase; PPIases accelerate the folding of proteins. It catalyzes the cis-trans isomerization of proline imidic peptide bonds in oligopeptides (216 aa)
PRDX1	Peroxiredoxin 1 (199 aa)
PSAT1	Phosphoserine aminotransferase 1 (370 aa)
PYGB	Alpha-1,4 glucan phosphorylase; Phosphorylase is an important allosteric enzyme in carbohydrate metabolism. Enzymes from different sources differ in their regulatory mechanisms and in their natural substrates. However, all known phosphorylases share catalytic and structural properties (843 aa)
RPS4	40S ribosomal protein S4, X isoform; Belongs to the eukaryotic ribosomal protein eS4 family (263 aa)
SEMA3C	Semaphorin 3C; Sema domain, immunoglobulin domain (Ig), short basic domain, secreted, (semaphorin) 3C; Belongs to the semaphorin family (751 aa)
Sep-7	Septin 7; Belongs to the TRAFAC class TrmE-Era-EngA-EngB-Septin- like GTPase superfamily. Septin GTPase family (384 aa)
SERPINC1	Serpin family C member 1; Serpin peptidase inhibitor, clade C (antithrombin), member 1; Belongs to the serpin family (464 aa)
SERPINE1	Serpin family E member 1; Serpin peptidase inhibitor, clade E (nexin, plasminogen activator inhibitor type 1), member 1; Belongs to the serpin family (402 aa)
SERPINF1	Serpin family F member 1; Serpin peptidase inhibitor, clade F (alpha-2 antiplasmin, pigment epithelium derived factor), member 1 (417 aa)
SERPINH1	Serpin family H member 1; Serpin peptiase inhibitor, clade H (heat shock protein 47), member 1, (collagen binding protein 1); Belongs to the serpin family (418 aa)
SERPINI1	Serpin family I member 1; Serpin peptidase inhibitor, clade I (neuroserpin), member 1; Belongs to the serpin family (410 aa)
SND1	Staphylococcal nuclease and tudor domain containing 1 (910 aa)

SPARC	Secreted protein, acidic, cysteine-rich (osteonectin) (333 aa)
STIP1	Stress-induced phosphoprotein 1 (541 aa)
TAGLN	Transgelin; Belongs to the calponin family (204 aa)
TAGLN2	Transgelin 2; Belongs to the calponin family (199 aa)
TARS	threonyl-tRNA synthetase (723 aa)
TCP1	T-complex 1; Molecular chaperone; assists the folding of proteins upon ATP hydrolysis (559 aa)
THBS2	Thrombospondin 2 (1172 aa)
THBS4	Thrombospondin 4 (983 aa)
TIMP2	TIMP metallopeptidase inhibitor 2 (221 aa)
TPM4	Tropomyosin 4; Belongs to the tropomyosin family (284 aa)
TUBB	Tubulin beta chain; Tubulin is the major constituent of microtubules. It binds two moles of GTP, one at an exchangeable site on the beta chain and one at a non-exchangeable site on the alpha chain (444 aa)
TUBB6	Tubulin beta chain; Tubulin is the major constituent of microtubules. It binds two moles of GTP, one at an exchangeable site on the beta chain and one at a non-exchangeable site on the alpha chain (446 aa)
UBA1	Ubiquitin-like modifier activating enzyme 1; Belongs to the ubiquitin-activating E1 family (1058 aa)
UGDH	UDP-glucose 6-dehydrogenase; Involved in the biosynthesis of glycosaminoglycans; hyaluronan, chondroitin sulfate, and heparan sulfate (494 aa)
UGP2	UDP-glucose pyrophosphorylase 2 (524 aa)
VASN	Vasorin (677 aa)
VCAN	Versican (3395 aa)
VIM	Vimentin; Belongs to the intermediate filament family (466 aa)
WARS	tryptophanyl-tRNA synthetase; Belongs to the class-I aminoacyl-tRNA synthetase family (476 aa)
YWHAE	Uncharacterized protein; Tyrosine 3-monooxygenase/tryptophan 5-monooxygenase activation protein, epsilon; Belongs to the 14-3-3 family (234 aa)
YWHAG	Tyrosine 3-monooxygenase/tryptophan 5-monooxygenase activation protein, gamma; Belongs to the 14-3-3 family (246 aa)

B. fAd-MSCs EXOSOMES	
**Abbreviation**	**Name**
ACO1	Aconitase 1, soluble; Belongs to the aconitase/IPM isomerase family (889 aa)
ACTB	Actin gamma 1; Belongs to the actin family (375 aa)
ACTC1	Actin, alpha, cardiac muscle 1; Belongs to the actin family (377 aa)
AFP	Alpha-fetoprotein (609 aa)
AKR1A1	Aldo-keto reductase family 1, member A1 (aldehyde reductase) (325 aa)
ALB	Serum albumin (607 aa)
ALDOA	Aldolase A, fructose-bisphosphate (701 aa)
ANXA11	Annexin A11 (505 aa)
ANXA4	Annexin A4 (319 aa)
ANXA6	Annexin A6 (673 aa)
ARPC1B	Actin-related protein 2/3 complex subunit; Functions as component of the Arp2/3 complex which is involved in regulation of actin polymerization and together with an activating nucleation-promoting factor (NPF) mediates the formation of branched actin networks (372 aa)
ASNS	Asparagine synthetase (glutamine-hydrolyzing) (561 aa)
ATIC	Uncharacterized protein (592 aa)
ATP2B4	Calcium-transporting ATPase; This magnesium-dependent enzyme catalyzes the hydrolysis of ATP coupled with the transport of calcium (1207 aa)
ATP5A1	ATP synthase subunit alpha; Produces ATP from ADP in the presence of a proton gradient across the membrane (553 aa)
ATP5B	ATP synthase subunit beta; Produces ATP from ADP in the presence of a proton gradient across the membrane (528 aa)
CAPG	Uncharacterized protein (349 aa)
CCDC80	Coiled-coil domain containing 80 (956 aa)
CCT2	Chaperonin containing TCP1 subunit 2; Molecular chaperone; assists the folding of proteins upon ATP hydrolysis (535 aa)
CCT3	T-complex protein 1 subunit gamma; Molecular chaperone; assists the folding of proteins upon ATP hydrolysis (577 aa)

CCT6A	Uncharacterized protein; Molecular chaperone; assists the folding of proteins upon ATP hydrolysis (531 aa)
CD151	Tetraspanin; CD151 molecule (Raph blood group) (252 aa)
CFB	Uncharacterized protein; Belongs to the peptidase S1 family (1268 aa)
CFL1	Uncharacterized protein; Belongs to the actin-binding proteins ADF family (223 aa)
CLIC1	Chloride intracellular channel 1; Belongs to the chloride channel CLIC family (241 aa)
CLK2	Secretory carrier-associated membrane protein; CDC-like kinase 2 (764 aa)
COL1A1	Collagen, type I, alpha 1 (1461 aa)
COL1A2	Collagen, type I, alpha 2 (1367 aa)
CTNNA1	Catenin alpha 1; Catenin (cadherin-associated protein), alpha 1, 102kDa (906 aa)
EEF1A1	Elongation factor 1-alpha 1; Belongs to the TRAFAC class translation factor GTPase superfamily. Classic translation factor GTPase family. EF-Tu/EF-1A subfamily (462 aa)
EEF1G	Eukaryotic translation elongation factor 1 gamma (490 aa)
EEF2	Uncharacterized protein; Eukaryotic translation elongation factor 2 (858 aa)
EHD2	EH-domain containing 2; Belongs to the TRAFAC class dynamin-like GTPase superfamily. Dynamin/Fzo/YdjA family (543 aa)
EHD3	EH-domain containing 3; Belongs to the TRAFAC class dynamin-like GTPase superfamily. Dynamin/Fzo/YdjA family (535 aa)
EIF4A1	Eukaryotic translation initiation factor 4A1; Belongs to the DEAD box helicase family (406 aa)
ENO1	Enolase 1, (alpha) (434 aa)
ENSFCAG00000007301	Adenosylhomocysteinase (432 aa)
ENSFCAG00000007862	annotation not available (342 aa)
ENSFCAG00000008652	60S ribosomal protein L18 (188 aa)
ENSFCAG00000012460	Adenylyl cyclase-associated protein ; Belongs to the CAP family (475 aa)
ENSFCAG00000012639	L-lactate dehydrogenase ; Belongs to the LDH/MDH superfamily (334 aa)
ENSFCAG00000015735	L-lactate dehydrogenase ; Belongs to the LDH/MDH superfamily (361 aa)
ENSFCAG00000025894	Uncharacterized protein; Belongs to the globin family (149 aa)

ENSFCAG00000025949	annotation not available (573 aa)
EZR	Ezrin (603 aa)
FGG	Fibrinogen gamma chain (437 aa)
FLNA	Filamin A, alpha (2651 aa)
FMOD	Fibromodulin (376 aa)
GDI2	Rab GDP dissociation inhibitor; Regulates the GDP/GTP exchange reaction of most RAB proteins by inhibiting the dissociation of GDP from them, and the subsequent binding of GTP (423 aa)
GJA1	Gap junction protein; One gap junction consists of a cluster of closely packed pairs of transmembrane channels, the connexons, through which materials of low MW diffuse from one cell to a neighboring cell (382 aa)
GNB2	G protein subunit beta 2; Guanine nucleotide binding protein (G protein), beta polypeptide 2 (340 aa)
GSN	Gelsolin (751 aa)
HBA	Hemoglobin subunit alpha; Involved in oxygen transport from the lung to the various peripheral tissues (142 aa)
HNRNPA2B1	Heterogeneous nuclear ribonucleoprotein A2/B1 (353 aa)
HSP90AA1	Heat shock protein 90kDa alpha (cytosolic), class A member 1 (745 aa)
HSP90AB1	Heat shock protein 90kDa alpha (cytosolic), class B member 1 (724 aa)
HSP90B1	Heat shock protein 90kDa beta (Grp94), member 1 (804 aa)
HSPA1L	Heat shock 70kDa protein 1-like (641 aa)
HSPA2	Heat shock 70kDa protein 2 (639 aa)
HSPA4	Heat shock 70kDa protein 4 (840 aa)
HSPA8	Heat shock 70kDa protein 8 (646 aa)
HSPA9	Heat shock 70kDa protein 9 (mortalin) (679 aa)
ITGB1	Integrin beta-1 (799 aa)
ITGB3	Integrin beta (783 aa)
ITIH2	Inter-alpha-trypsin inhibitor heavy chain 2 (950 aa)
K-RAS	KRAS proto-oncogene, GTPase (188 aa)

KRT1	Keratin 1; Belongs to the intermediate filament family (630 aa)
KRT2	Keratin 2; Belongs to the intermediate filament family (666 aa)
KRT76	Keratin 76; Belongs to the intermediate filament family (617 aa)
LAMB1	Laminin, beta 1 (1786 aa)
LGALS1	Galectin; Lectin, galactoside-binding, soluble, 1 (135 aa)
LGALS3	Galectin; Lectin, galactoside-binding, soluble, 3 (278 aa)
LOXL2	Lysyl oxidase-like 2 (773 aa)
LUM	Lumican (339 aa)
MSN	Moesin (577 aa)
N-RAS	NRAS proto-oncogene, GTPase (189 aa)
NRP1	Neuropilin 1; Belongs to the neuropilin family (924 aa)
PGAM4	Phosphoglycerate mutase family member 4; Belongs to the phosphoglycerate mutase family. BPG- dependent PGAM subfamily (254 aa)
PGD	6-phosphogluconate dehydrogenase, decarboxylating; Belongs to the 6-phosphogluconate dehydrogenase family (483 aa)
PGK1	Phosphoglycerate kinase (417 aa)
PGK2	Phosphoglycerate kinase 2 (417 aa)
PPIB	Peptidyl-prolyl cis-trans isomerase; PPIases accelerate the folding of proteins. It catalyzes the cis-trans isomerization of proline imidic peptide bonds in oligopeptides (216 aa)
PRDX1	Peroxiredoxin 1 (199 aa)
PSAT1	Phosphoserine aminotransferase 1 (370 aa)
RAB10	RAB10, member RAS oncogene family (200 aa)
RAB11B	RAB11B, member RAS oncogene family (222 aa)
RAB14	RAB14, member RAS oncogene family (215 aa)
RAB1A	RAB1A, member RAS oncogene family (205 aa)
RAB1B	RAB1B, member RAS oncogene family (201 aa)

RAB2B	RAB2B, member RAS oncogene family (216 aa)
RAB5A	RAB5A, member RAS oncogene family (240 aa)
RAB5B	RAB5B, member RAS oncogene family (243 aa)
RAB5C	RAB5C, member RAS oncogene family (216 aa)
RALA	RAS like proto-oncogene A; V-ral simian leukemia viral oncogene homolog A (ras related) (206 aa)
RAP1A	RAP1A, member of RAS oncogene family (184 aa)
RAP1B	RAP1B, member of RAS oncogene family (184 aa)
RFTN1	Raftlin, lipid raft linker 1 (555 aa)
RPS4	40S ribosomal protein S4, X isoform; Belongs to the eukaryotic ribosomal protein eS4 family (263 aa)
SERPINC1	Serpin family C member 1; Serpin peptidase inhibitor, clade C (antithrombin), member 1; Belongs to the serpin family (464 aa)
SERPINE1	Serpin family E member 1; Serpin peptidase inhibitor, clade E (nexin, plasminogen activator inhibitor type 1), member 1; Belongs to the serpin family (402 aa)
SERPINH1	Serpin family H member 1; Serpin peptidase inhibitor, clade H (heat shock protein 47), member 1, (collagen binding protein 1); Belongs to the serpin family (418 aa)
SLC2A1	Solute carrier family 2 member 1; Belongs to the major facilitator superfamily. Sugar transporter (TC 2.A.1.1) family (492 aa)
SLC3A2	Solute carrier family 3 (amino acid transporter heavy chain), member 2 (533 aa)
STOM	Stomatin (284 aa)
TAGLN	Transgelin; Belongs to the calponin family (204 aa)
TAGLN2	Transgelin 2; Belongs to the calponin family (199 aa)
TCP1	T-complex 1; Molecular chaperone; assists the folding of proteins upon ATP hydrolysis (559 aa)
TUBA4A	Tubulin alpha chain; Tubulin is the major constituent of microtubules. It binds two moles of GTP, one at an exchangeable site on the beta chain and one at a non-exchangeable site on the alpha chain (482 aa)
TUBB	Tubulin beta chain; Tubulin is the major constituent of microtubules. It binds two moles of GTP, one at an exchangeable site on the beta chain and one at a non-exchangeable site on the alpha chain (444 aa)
TUBB6	Tubulin beta chain; Tubulin is the major constituent of microtubules. It binds two moles of GTP, one at an

	exchangeable site on the beta chain and one at a non-exchangeable site on the alpha chain (446 aa)
UBA1	Ubiquitin-like modifier activating enzyme 1; Belongs to the ubiquitin-activating E1 family (1058 aa)
UGDH	UDP-glucose 6-dehydrogenase; Involved in the biosynthesis of glycosaminoglycans; hyaluronan, chondroitin sulfate, and heparan sulfate (494 aa)
UGP2	UDP-glucose pyrophosphorylase 2 (524 aa)
VCAN	Versican (3395 aa)
VIM	Vimentin; Belongs to the intermediate filament family (466 aa)
WARS	tryptophanyl-tRNA synthetase; Belongs to the class-I aminoacyl-tRNA synthetase family (476 aa)
YWHAE	Uncharacterized protein; Belongs to the 14-3-3 family (234 aa)
YWHAG	Tyrosine 3-monooxygenase/tryptophan 5-monooxygenase activation protein, gamma; Belongs to the 14-3-3 family (246 aa)
