# Supplementary material for: Proteomic Analysis of the Secretome and Exosomes of Feline Adipose-Derived Mesenchymal Stem Cells
Source: Animals (Basel). 2021 Jan 24;11(2):295. doi: 10.3390/ani11020295 (PMC7912403; doi:10.3390/ani11020295)
Supplement: Supplementary file 1 [file animals-11-00295-s001.zip › S3 Table. Complete list of KEGG pathways in secretome and exosome proteomes..pdf]

S3 Table. Complete list of KEGG pathways in secretome and exosome proteomes.

A. fAd-MSC SECRETOME					
**Number**	**Term ID**	**Term Description**	**Observed Gene count**	**False Discovery Rate**	**Proteins**
1	fca01100	Metabolic pathways	21	0.00029	AKR1A1; ALDOA; ALDOC; ATIC; ENO1; ENSFCAG00000007301; ENSFCAG00000012639; ENSFCAG00000015735; GANAB; GAPD; HEXB; IDH1; LAP3; MDH1; PGAM4; PGD; PGK1; PSAT1; PYGB; UGDH; UGP2
2	fca01200	Carbon metabolism	10	2.99e-07	ALDOA; ALDOC; ENO1; GAPD; IDH1; MDH1; PGAM; PGD; PGK1; PSAT1
3	fca04151	PI3K-Akt signaling pathway	10	0.00060	COL1A2; COMP; HSP90AA1; HSP90AB1; HSP90B1; LAMB1; THBS2; THBS4; YWHAE; YWHAG
4	fca00010	Glycolysis / Gluconeogenesis	9	6.48e-08	AKR1A1; ALDOA; ALDOC; ENO1; ENSFCAG00000012639; ENSFCAG00000015735; GAPD; PGAM4; PGK1
5	fca01230	Biosynthesis of amino acids	8	1.17e-06	ALDOA; ALDOC; ENO1; GAPD; IDH1; PGAM4; PGK1; PSAT1
6	fca04141	Protein processing in endoplasmic reticulum	8	0.00019	GANAB; HSP90AA1; HSP90AB1; HSP90B1; HSPA1L; HSPA8; PDIA3; PDIA6
7	fca04510	Focal adhesion	8	0.00053	ACTB; ACTN1; COL1A2; COMP; FLNA; LAMB1; THBS2; THBS4
8	fca04612	Antigen processing and presentation	7	7.07e-06	CTSB; HSP90AA1; HSP90AB1; HSPA1L; HSPA4; HSPA8; PDIA3
9	fca05169	Epstein-Barr virus infection	7	0.0020	HSPA1L; HSPA8; HSPB1; SND1; VIM; YWHAE; YWHAG
10	fca05205	Proteoglycans in cancer	7	0.0023	ACTB; DCN; EZR; FLNA; LUM; MMP2; MSN
11	fca04145	Phagosome	6	0.0023	ACTB; COMP; THBS2; THBS4; TUBB; TUBB6

12	fca04915	Estrogen signaling pathway	6	0.0023	HSP90AA1; HSP90AB1; HSP90B1; HSPA1L; HSPA8; MMP2
13	fca05203	Viral carcinogenesis	6	0.0050	ACTN1; GSN; HIST1H2BB; SND1; YWHAE; YWHAG
14	fca04810	Regulation of actin cytoskeleton	6	0.0083	ACTB; ACTN1; ARPC1B; EZR; GSN; MSN
15	fca04512	ECM-receptor interaction	5	0.0023	COL1A2; COMP; LAMB1; THBS2; THBS4
16	fca05146	Amoebiasis	5	0.0023	ACTN1; COL1A2; COL3A1; HSPB1; LAMB1
17	fca04670	Leukocyte transendothelial migration	5	0.0039	ACTB; ACTN1; EZR; MMP2; MSN
18	fca05418	Fluid shear stress and atherosclerosis	5	0.0084	ACTB; HSP90AA1; HSP90AB1; HSP90B1; MMP2
19	fca04390	Hippo signaling pathway	5	0.0097	ACTB; AFP; SERPINE1; YWHAE; YWHAG
20	fca04530	Tight junction	5	0.0153	ACTB; ACTN1; EZR; HSPA4; MSN
21	fca00270	Cysteine and methionine metabolism	4	0.0023	ENSFCAG00000007301; ENSFCAG00000012639; ENSFCAG00000015735; MDH1
22	fca05134	Legionellosis	4	0.0023	EEF1A1; EEF1G; HSPA1L; HSPA8
23	fca05144	Malaria	4	0.0023	COMP; HBA; THBS2; THBS4
24	fca04933	AGE-RAGE signaling pathway in diabetic complications	4	0.0151	COL1A2; COL3A1; MMP2; SERPINE1
25	fca04922	Glucagon signaling pathway	4	0.0153	ENSFCAG00000012639; ENSFCAG00000015735; PGAM4; PYGB
26	fca04926	Relaxin signaling pathway	4	0.0309	ACTA2; COL1A2; COL3A1; MMP2
27	fca00040	Pentose and glucuronate interconversions	3	0.0023	AKR1A1; UGDH; UGP2
28	fca00030	Pentose phosphate pathway	3	0.0061	ALDOA; ALDOC; PGD

29	fca00620	Pyruvate metabolism	3	0.0097	ENSFCAG00000012639; ENSFCAG00000015735; MDH1
30	fca00480	Glutathione metabolism	3	0.0157	IDH1; LAP3; PGD
31	fca00520	Amino sugar and nucleotide sugar metabolism	3	0.0180	HEXB; UGDH; UGP2

B. fAd-MSC EXOSOMES					
**Number**	**Term ID**	**Term Description**	**Observed Gene Count**	**False Discovery Rate**	**Proteins**
1	fca01100	Metabolic pathways	**18**	0.0013	ACO1; AKR1A1; ALDOA; ASNS; ATIC; ATP5A1; ATP5B; ENO1; ENSFCAG00000007301; ENSFCAG00000012639; ENSFCAG00000015735; PGAM4; PGD; PGK1; PGK2; PSAT1; UGDH; UGP2
2	fca04151	PI3K-Akt signaling pathway	**13**	4.59e-06	COL1A1; COL1A2; GNB2; HSP90AA1; HSP90AB1; HSP90B1; ITGB1; ITGB3; K-RAS; LAMB1; N-RAS; YWHAE; YWHAG
3	fca04144	Endocytosis	**11**	7.15e-06	ARPC1B; EHD2; EHD3; HSPA1L; HSPA2; HSPA8; RAB10; RAB11B; RAB5A; RAB5B; RAB5C
4	fca05200	Pathways in cancer	**11**	0.00093	CTNNA1; GNB2; HSP90AA1; HSP90AB1; HSP90B1; ITGB1; K-RAS; LAMB1; N-RAS; RALA; SLC2A1
5	fca04145	Phagosome	**9**	7.15e-06	ACTB; ITGB1; ITGB3; RAB5A; RAB5B; RAB5C; TUBA4A; TUBB; TUBB6;
6	fca05205	Proteoglycans in cancer	**9**	3.72e-05	ACTB; EZR; FLNA; ITGB1; ITGB3; K-RAS; LUM; MSN; N-RAS
7	fca04510	Focal adhesion	**9**	3.86e-05	ACTB; COL1A1; COL1A2; FLNA; ITGB1; ITGB3; LAMB1; RAP1A; RAP1B
8	fca04810	Regulation of actin cytoskeleton	**9**	4.19e-05	ACTB; ARPC1B; EZR; GSN; ITGB1; ITGB3; K-RAS; MSN; N-RAS
9	fca04014	Ras signaling pathway	**9**	6.89e-05	GNB2; K-RAS; N-RAS; RAB5A; RAB5B; RAB5C; RALA; RAP1A; RAP1B
10	fca05134	Legionellosis	**8**	2.35e-07	EEF1A1; EEF1G; ENSFCAG00000025949; HSPA1L; HSPA2; HSPA8; RAB1A; RAB1B
11	fca00010	Glycolysis / Gluconeogenesis	**8**	6.69e-07	AKR1A1; ALDOA; ENO1; ENSFCAG00000012639; ENSFCAG00000015735; PGAM4; PGK1; PGK2

12	fca04611	Platelet activation	**8**	1.13e-05	ACTB; COL1A1; COL1A2; FGG; ITGB1; ITGB3; RAP1A; RAP1B
13	fca01200	Carbon metabolism	**8**	1.29e-05	ACO1; ALDOA; ENO1; PGAM4; PGD; PGK1; PGK2; PSAT1
14	fca04915	Estrogen signaling pathway	**8**	2.85e-05	HSP90AA1; HSP90AB1; HSP90B1; HSPA1L; HSPA2; HSPA8; K-RAS; N-RAS
15	fca04015	Rap1 signaling pathway	**8**	0.00025	ACTB; ITGB1; ITGB3; K-RAS; N-RAS; RALA; RAP1A; RAP1B
16	fca04010	MAPK signaling pathway	**8**	0.0014	FLNA; HSPA1L; HSPA2; HSPA8; K-RAS; N-RAS; RAP1A; RAP1B
17	fca01230	Biosynthesis of amino acids	**7**	1.02e-05	ACO1; ALDOA; ENO1; PGAM4; PGK1; PGK2; PSAT1
18	fca04670	Leukocyte transendothelial migration	**7**	5.21e-05	ACTB; CTNNA1; EZR; ITGB1; MSN; RAP1A; RAP1B
19	fca04530	Tight junction	**7**	0.00044	ACTB; EZR; HSPA4; ITGB1; MSN; RAP1A; TUBA4A
20	fca05165	Human papillomavirus infection	**7**	0.0070	COL1A1; COL1A2; ITGB1; ITGB3; K-RAS; LAMB1; N-RAS
21	fca04612	Antigen processing and presentation	**6**	4.19e-05	HSP90AA1; HSP90AB1; HSPA1L; HSPA2; HSPA4; HSPA8
22	fca04540	Gap junction	**6**	9.88e-05	GJA1; K-RAS; N-RAS; TUBA4A; TUBB; TUBB6
23	fca05146	Amoebiasis	**6**	0.00024	COL1A1; COL1A2; LAMB1; RAB5A; RAB5B; RAB5C
24	fca04390	Hippo signaling pathway	**6**	0.0013	ACTB; AFP; CTNNA1; SERPINE1; YWHAE; YWHAG
25	fca04141	Protein processing in endoplasmic reticulum	**6**	0.0021	HSP90AA1; HSP90AB1; HSP90B1; HSPA1L; HSPA2; HSPA8
26	fca05169	Epstein-Barr virus infection	**6**	0.0035	HSPA1L; HSPA2; HSPA8; VIM; YWHAE; YWHAG
27	fca04213	Longevity regulating pathway - multiple species	**5**	0.00029	HSPA1L; HSPA2; HSPA8; K-RAS; N-RAS

28	fca05211	Renal cell carcinoma	**5**	0.00052	K-RAS; N-RAS; RAP1A; RAP1B; SLC2A1
29	fca05412	Arrhythmogenic right ventricular cardiomyopathy (ARVC)	**5**	0.00052	ACTB; CTNNA1; GJA1; ITGB1; ITGB3
30	fca04512	ECM-receptor interaction	**5**	0.0010	COL1A1; COL1A2; ITGB1; ITGB3; LAMB1
31	fca05215	Prostate cancer	**5**	0.0014	HSP90AA1; HSP90AB1; HSP90B1; K-RAS; N-RAS
32	fca04933	AGE-RAGE signaling pathway in diabetic complications	**5**	0.0016	COL1A1; COL1A2; K-RAS; N-RAS; SERPINE1
33	fca05145	Toxoplasmosis	**5**	0.0022	HSPA1L; HSPA2; HSPA8; ITGB1; LAMB1
34	fca04919	Thyroid hormone signaling pathway	**5**	0.0025	ACTB; ITGB3; K-RAS; N-RAS; SLC2A1
35	fca04722	Neurotrophin signaling pathway	**5**	0.0035	K-RAS; N-RAS; RAP1A; RAP1B; YWHAE
36	fca04926	Relaxin signaling pathway	**5**	0.0040	COL1A1; COL1A2; GNB2; K-RAS; N-RAS
37	fca05418	Fluid shear stress and atherosclerosis	**5**	0.0051	ACTB; HSP90AA1; HSP90AB1; HSP90B1; ITGB3
38	fca05206	MicroRNAs in cancer	**5**	0.0054	EZR; ITGB3; K-RAS; N-RAS; VIM
39	fca04062	Chemokine signaling pathway	**5**	0.0103	GNB2; K-RAS; N-RAS; RAP1A; RAP1B
40	fca05152	Tuberculosis	**5**	0.0103	ENSFCAG00000025949; HSPA9; RAB5A; RAB5B; RAB5C
41	fca05203	Viral carcinogenesis	**5**	0.0130	GSN; K-RAS; N-RAS; YWHAE; YWHAG
42	fca04714	Thermogenesis	**5**	0.0292	ACTB; ATP5A1; ATP5B; K-RAS; N-RAS
43	fca04962	Vasopressin-regulated water reabsorption	**4**	0.0011	RAB11B; RAB5A; RAB5B; RAB5C

44	fca05230	Central carbon metabolism in cancer	**4**	0.0023	K-RAS; N-RAS; PGAM4; SLC2A1
45	fca04720	Long-term potentiation	**4**	0.0034	K-RAS; N-RAS; RAP1A; RAP1B
46	fca05100	Bacterial invasion of epithelial cells	**4**	0.0049	ACTB; ARPC1B; CTNNA1; ITGB1
47	fca05410	Hypertrophic cardiomyopathy (HCM)	**4**	0.0051	ACTB; ACTC1; ITGB1; ITGB3
48	fca05414	Dilated cardiomyopathy (DCM)	**4**	0.0053	ACTB; ACTC1; ITGB1; ITGB3
49	fca04610	Complement and coagulation cascades	**4**	0.0064	CFB; FGG; SERPINC1; SERPINE1
50	fca04922	Glucagon signaling pathway	**4**	0.0103	ENSFCAG0000001263; ENSFCAG0000001573; PGAM4; SLC2A1
51	fca05162	Measles	**4**	0.0150	HSPA1L; HSPA2; HSPA8; MSN
52	fca04152	AMPK signaling pathway	**4**	0.0195	EEF2; RAB10; RAB11B; RAB14
53	fca04210	Apoptosis	**4**	0.0215	ACTB; K-RAS; N-RAS; TUBA4A
54	fca04371	Apelin signaling pathway	**4**	0.0249	GNB2; K-RAS; N-RAS; SERPINE1
55	fca04921	Oxytocin signaling pathway	**4**	0.0303	ACTB; EEF2; K-RAS; N-RAS
56	fca05164	Influenza A	**4**	0.0335	ACTB; HSPA1L; HSPA2; HSPA8
57	fca04360	Axon guidance	**4**	0.0403	ITGB1; K-RAS; N-RAS; NRP1
58	fca00040	Pentose and glucuronate interconversions	**3**	0.0011	AKR1A1; UGDH; UGP2

59	fca00270	Cysteine and methionine metabolism	**3**	0.0103	ENSFCAG0000000730; ENSFCAG0000001263; ENSFCAG00000015735
60	fca05213	Endometrial cancer	**3**	0.0147	CTNNA1; K-RAS; N-RAS
61	fca03018	RNA degradation	**3**	0.0321	ENO1; ENSFCAG00000025949; HSPA9
62	fca04974	Protein digestion and absorption	**3**	0.0327	COL1A1; COL1A2; SLC3A2
63	fca05132	Salmonella infection	**3**	0.0334	ACTB; ARPC1B; FLNA
64	fca05210	Colorectal cancer	**3**	0.0335	K-RAS; N-RAS; RALA
65	fca04972	Pancreatic secretion	**3**	0.0389	ATP2B4; RAP1A; RAP1B
66	fca04657	IL-17 signaling pathway	**3**	0.0422	HSP90AA1; HSP90AB1; HSP90B1
67	fca04914	Progesterone-mediated oocyte maturation	**3**	0.0422	HSP90AA1; HSP90AB1; K-RAS
68	fca00030	Pentose phosphate pathway	**2**	0.0390	ALDOA; PGD
69	fca00640	Propanoate metabolism	**2**	0.0440	ENSFCAG00000012639; ENSFCAG00000015735
70	fca05216	Thyroid cancer	**2**	0.0459	K-RAS; N-RAS
