# Supplementary figures and images for: Proteomic Analysis of the Secretome and Exosomes of Feline Adipose-Derived Mesenchymal Stem Cells
Source: Animals (Basel). 2021 Jan 24;11(2):295. doi: 10.3390/ani11020295 (PMC7912403; doi:10.3390/ani11020295)

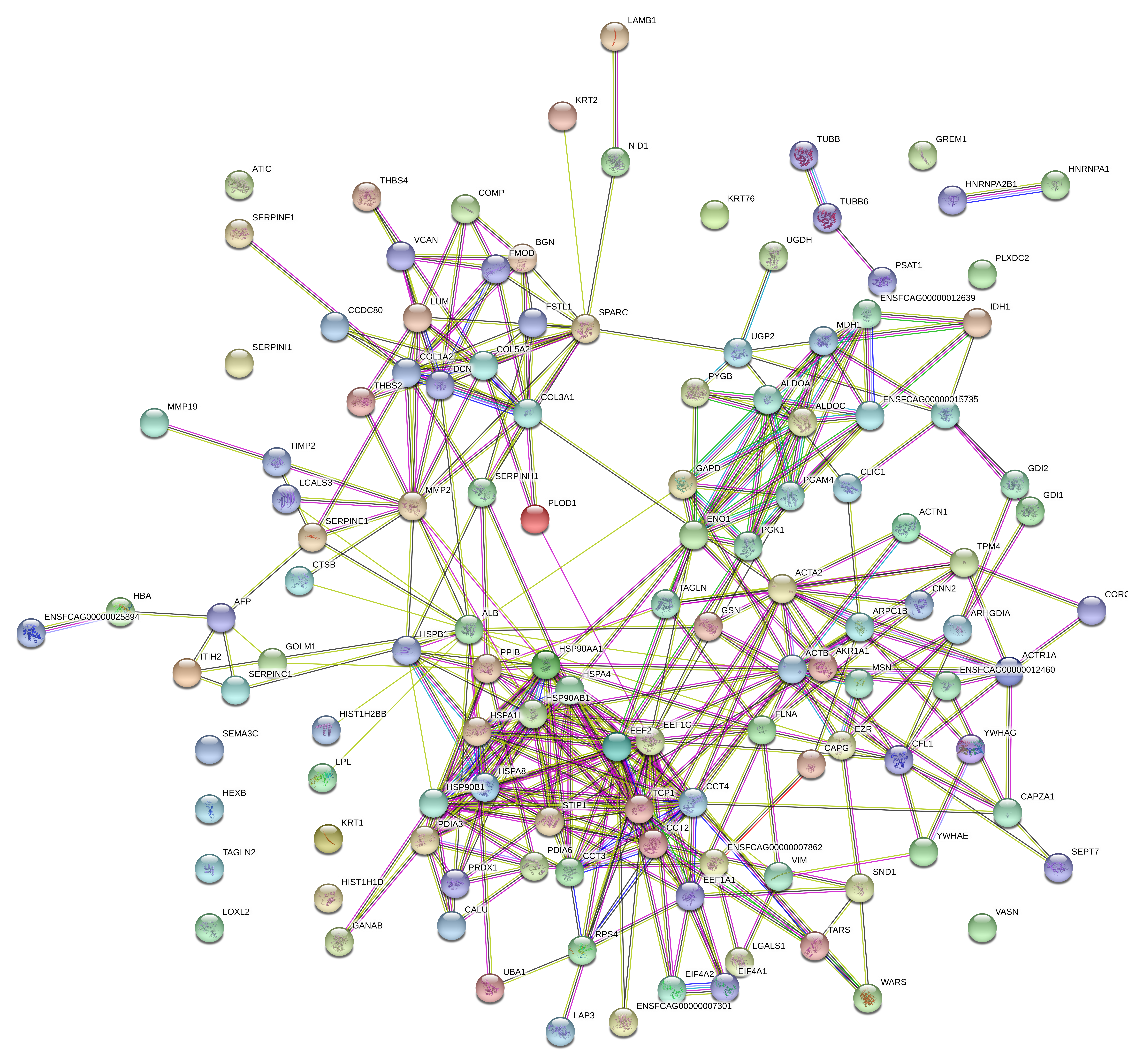

Supplement: Supplementary file 1 [file animals-11-00295-s001.zip › S1 Figure_Network of proteins identified in the fAd-MSCs secretome.png]

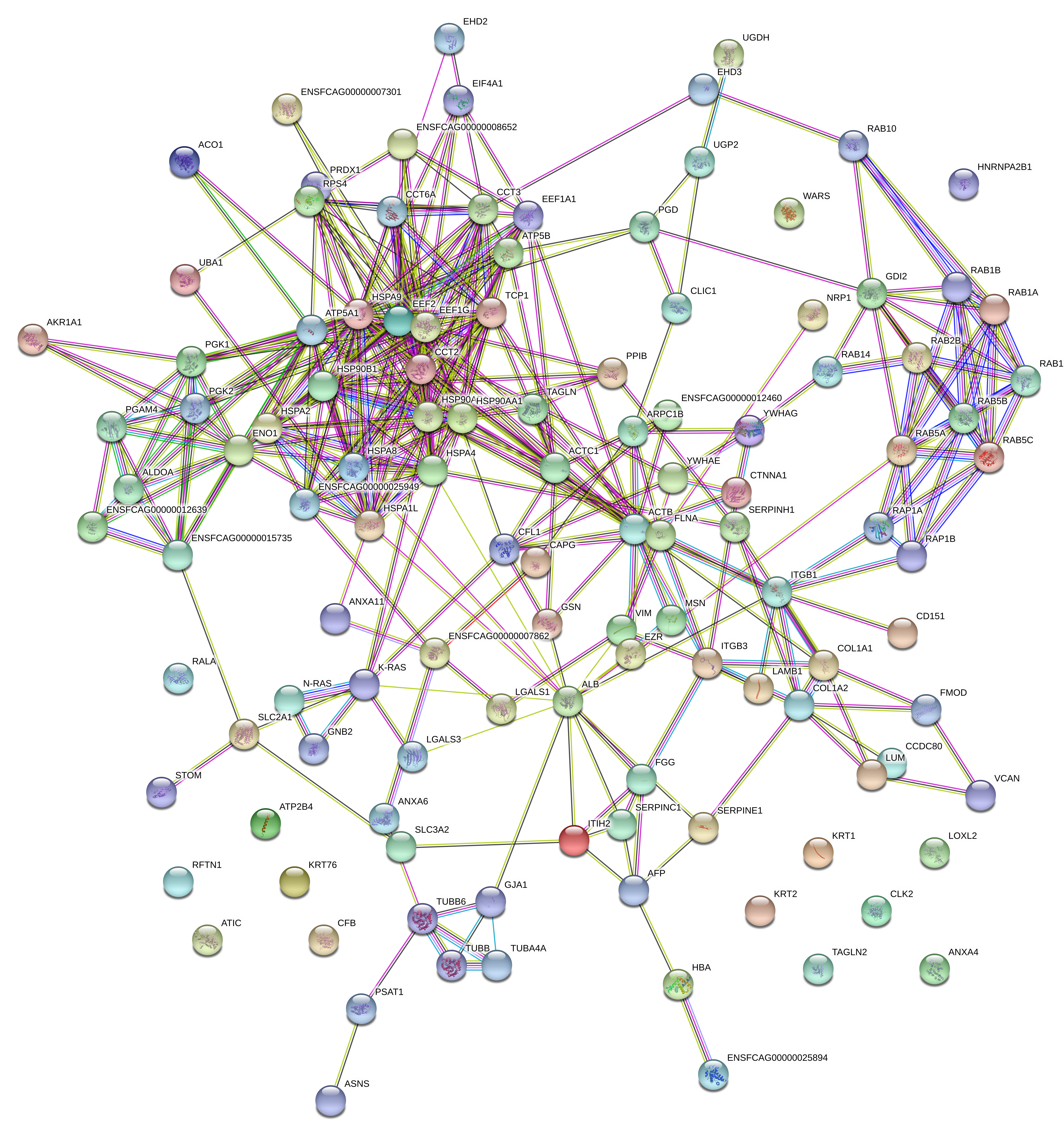

Supplement: Supplementary file 1 [file animals-11-00295-s001.zip › S2 Figure_Network of proteins identified in the fAd-MSCs exosomes.png]
